# Thermosensitive Hydrogel Sustaining the Release of Lymph-Draining Oligonucleotide Adjuvant Polyplex Micelles Improves Systemic Cancer Immunotherapy

**DOI:** 10.1021/acsnano.5c05517

**Published:** 2025-06-03

**Authors:** Samuel N. Lucas, Paul A. Archer, Tae Hee Yoon, Margaret P. Manspeaker, Maya Levitan, Jihoon Kim, Susan N. Thomas

**Affiliations:** † Wallace H. Coulter Department of Biomedical Engineering, Georgia Institute of Technology and Emory University, Atlanta, Georgia 30332, United States; ‡ Parker H. Petit Institute for Bioengineering and Bioscience, 1372 Georgia Institute of Technology, Atlanta, Georgia 30332, United States; § School of Chemical and Biomolecular Engineering, Georgia Institute of Technology, Atlanta, Georgia 30332, United States; ∥ George W. Woodruff School of Mechanical Engineering, Georgia Institute of Technology, Atlanta, Georgia 30332, United States; ⊥ School of Integrative Engineering, 26729 Chung-Ang University, Seoul 06974, South Korea; # Winship Cancer Institute, Emory University, Atlanta, Georgia 30322, United States

**Keywords:** immunotherapy, sustained release, lymphatic delivery, immune checkpoint blockade, lymph node

## Abstract

Immune checkpoint blockade (ICB) immunotherapies are a powerful tool in the clinical management of cancer, but response rates to ICB remain limited, and treatment-related toxicities can be significant. Therapeutic efficacy of ICB can be enhanced by delivering synergistic immunomodulators to tumor-draining lymph nodes (TdLNs). However, achieving sustained release of small molecule immunomodulators into the lymphatics and TdLNs remains challenging. To address this limitation, a sustained release system for delivering an oligonucleotide adjuvant to lymph nodes (LNs) was developed. CpG oligonucleotide was complexed with a redox-responsive cationic polymer and mixed with F127-*g*-Gelatin to generate a thermosensitive hydrogel that releases lymph-draining polyplex micelles *in situ*. This CpG/BPEI-SS-/F127-*g*-Gelatin (CpG-HG) system enhanced the quantity and duration of CpG delivery to TdLNs following locoregional administration compared with free drug and enabled targeted, potent, and prolonged immunomodulation within TdLNs from a single administration. This augmented, localized immune response synergized with systemic ICB treatment, both markedly amplifying the systemic circulating CD8+ T cell response and improving antitumor therapeutic efficacy while enabling ICB dose reduction. These results highlight the potential for this drug delivery system as an adjunct to existing clinical ICB protocols to improve patient outcomes.

## Graphical abstract

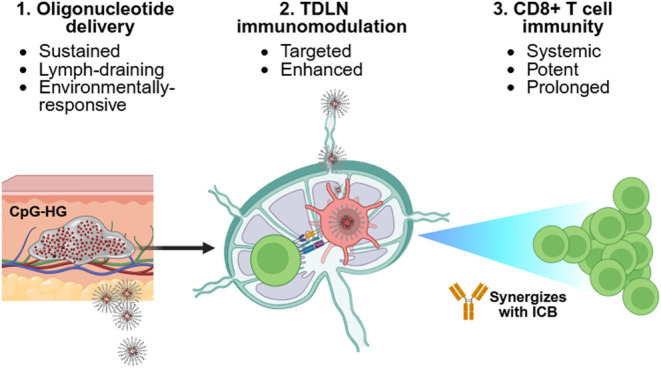

## Introduction

The emergence of cancer immunotherapies that modulate the antitumor immune response to reestablish tumor control has transformed the clinical management of cancer. In particular, immune checkpoint blockade (ICB) which leverages antagonistic antibodies against immune checkpoints programmed cell death protein 1 (PD-1), cytotoxic T lymphocyte associated protein 4 (CTLA-4), and programmed death ligand 1 (PD-L1) has seen significant success and widespread adoption in a variety of cancer types and stages.^1–4^ Despite this, response rates to ICB remain limited. Therapeutic approaches combining ICB with other immunomodulatory agents have been investigated as one strategy to potentially improve ICB efficacy with promising preclinical results.^5,6^ However, with the exception of combinations of ICB antibodies, these methods have yet to see significant clinical success, suggesting that conventional administration of multiple immunomodulatory agents to a cancer patient may be insufficient to achieve therapeutic efficacy without incurring unacceptable toxicity.

Increasingly, there is a growing understanding that lymph nodes (LNs), particularly sentinel LNs (SLNs) and other tumor-draining LNs (TdLNs), are sites that enable localized immune education capable of generating systemically active and functional immune responses.^7–9^ These LNs bring together T cells, tumor antigen, and resident and migratory antigen presenting cells (APCs) such as dendritic cells (DCs), and they are critical sites for tumor-reactive T cell priming and expansion. Beyond the initiation and maintenance of the antitumor adaptive immune response generally, a growing body of research has implicated TdLNs in enabling response to ICB as well as other immune modulators including pattern recognition receptor (PRR) agonists.^10–16^ While TdLNs therefore hold potential as therapeutic targets, current clinical paradigms of immunotherapy rarely seek to achieve therapeutic TdLN targeting.^17^

The importance of TdLNs in enabling the response to cancer immunotherapy motivates the design and application of engineered drug delivery systems to improve immunotherapeutic delivery to TdLNs. Because systemically administered therapies have limited access to the lymphatic system, locoregional administration is an attractive approach to increase drug accumulation in TdLNs. While therapeutic IgG antibodies are sufficiently large to facilitate lymphatic drainage following intradermal (i.d.) or subcutaneous injection, smaller drugs require drug delivery systems both to enable appreciable lymphatic delivery and to reduce systemic exposure resulting from rapid clearance from the site of injection via blood absorption.^10,18–21^ One conceptual approach to addressing this problem involves using nanocarrier systems to deliver chemically conjugated or physically absorbed small molecules or oligonucleotides to the TdLN following locoregional administration.^14,22–24^ Such nanocarrier systems can achieve lymphatic delivery but may exhibit limited sustained release and consequently may require repeated administration: for example, 30 nm diameter polypropylene sulfide nanoparticles achieve efficient LN delivery but over 90% of the injected nanoparticles are cleared from the injection site within 24 h after intradermal injection.^25^ Conventional sustained release systems, on the other hand, may face difficulty achieving efficient lymphatic uptake in the context of smaller drugs due to rapid absorption of the drug into the circulation after drug release. These challenges are heightened when considering the delivery of therapeutic oligonucleotides, which are additionally subject to class-specific delivery challenges such as degradation by nucleases.^26^ In contrast, a drug delivery system capable of enabling sustained drug release of small molecules or oligonucleotides into the lymphatic system has potential to maintain therapeutically relevant drug concentrations in LNs while at the same time mitigating systemic toxicities by reducing the number of administrations necessary for therapeutic benefit.^27^

Here, we report a locoregional oligonucleotide adjuvant delivery system that combines advantages presented by both sustained release systems and nanocarrier systems to enhance and prolong the delivery of oligonucleotide to TdLNs, provoking local immunomodulation that can be exploited using ICB to generate systemic immune responses leading to tumor control. As a model oligonucleotide adjuvant, we utilize the toll-like receptor 9 (TLR9) agonist CpG oligonucleotide, In APCs, TLR9 ligation by CpG initiates NF-kB, AP1, and IRF-7 signaling which in turn results in upregulation of immunostimulatory factors including costimulatory molecules (e.g., CD86) and proinflammatory cytokines (e.g., type 1 interferon).^28–31^ To achieve CpG delivery, disulfide-cross-linked polyethylenimine (BPEI-SS-) that exhibits high transfection efficacy and low cytotoxicity was prepared from low molecular weight BPEI, facilitating the formation of polyplexes with CpG via electrostatic interactions.^32–34^ F127-grafted gelatin polymer (F127-*g*-Gelatin) exhibiting anionic surface charge was employed for further electrostatic interactions with the CpG/BPEI-SS- polyplex, optimizing the size and surface charge of CpG-loaded nanocarrier systems for efficient lymphatic uptake. F127-*g*-Gelatin is a biocompatible and biodegradable polymer developed by our group recently, which not only behaves as a thermosensitive hydrogel but also sustains the release of lymph-draining micelles.^35^ Simple mixing of CpG/BPEI-SS- polyplexes into an F127-*g*-Gelatin solution resulted in the formation of thermosensitive hydrogels that release nanoscale CpG/BPEI-SS-/F127-*g*-Gelatin (CpG-HG) polyplex micelles appropriate for efficient lymph drainage and sustained lymph node accumulation. When administered locoregionally, CpG-HG sustained delivery of CpG oligonucleotide to antigen presenting cells (APCs) in TdLNs, the mechanism of which differing by APC subtype, prolonging DC activation. As a result, T cell expansion both locally in LNs draining the CpG injection site as well as in the circulation was both remarkably increased as well as prolonged, the latter for as long as 9 days post a single treatment. Moreover, a single CpG-HG treatment not only augmented CpG synergies with ICB but also achieved ICB dose sparing. This demonstrates the potential for this lymph-targeting drug delivery system as a potential adjunct to current systemic ICB therapeutic paradigms.

## Results and Discussion

### Cell Mobilization from the TdLN Is Critical for Tumor Control and Is Enhanced by Immunotherapy

Recent work by several groups has demonstrated that to a substantial degree the response to immunotherapies such as ICB occurs outside of the tumor microenvironment (TME) and that intratumoral T cells are replaced by new infiltrating T cells originating from other tissues.^36,37^ To evaluate the importance of the TdLN as a source of T cells for mobilization and tumor control, B16F10 tumor-bearing mice were intraperitoneally (i.p.) treated with either the sphingosine 1-phosphate (S1P) receptor modulator FTY720 (FTY), which inhibits lymphocyte egress from LNs, or DMSO in saline (vehicle) control 7 and 9 days after tumor implantation (Figure S1). 24 h after treatment, both CD8+ and CD4+ T cells were observed to be significantly decreased in the blood for FTY treated mice compared to those receiving the vehicle control (Figure 1A). This reduction in circulating T cells resulted in more rapid tumor growth for FTY treated mice and poorer survival compared to vehicle treated mice, highlighting the importance of LNs for supplying circulating T cells for tumor control (Figure 1B,C). Because FTY inhibits lymphocyte egress from LNs independently of anatomical location, transgenic mice expressing the photoactivatable fluorescent protein PA-GFP in their hematopoietic cells were additionally used to more specifically examine the contribution of the TdLN in supplying T cells to the TME (Figure 1D). PA-GFP is a mutant form of GFP that is not fluorescent in its base form but becomes fluorescent upon exposure to 405 nm light (photoactivation).^38^ Upon photoactivation of an LN in a PA-GFP mouse, the lymphocytes within that LN become fluorescent (Figure 1E). Subsequently, tissues of interest can be analyzed using flow cytometry to identify the fluorescence signature of PA-GFP with the knowledge that photoactivated (PA-GFP+) cells must have been in the target LN at time of photoactivation and subsequently migrated to the analyzed tissue. TdLNs in B16F10 tumor-bearing mice were photoactivated in this manner and the tumors were analyzed 24 h after LN photoactivation. To confirm that inadvertent photolabeling of cells in the TME did not occur, some mice were euthanized 5 min after LN photoactivation and the PA-GFP signal in CD8+ T cells in those tumors were compared to the PA-GFP signal in CD8+ T cells in tumors of mice that did not receive TdLN photoactivation (Figure 1F). 24 h after TdLN photoactivation, PA-GFP+ CD8+ T cells were observed in the TME, indicating that the TdLN supplies T cells for tumor control (Figure 1G). Furthermore, when mice received antibodies against the immune checkpoints PD-1 and CTLA-4 (ICB) via systemic (i.p.) administration, the frequency of PA-GFP+ CD8+ T cells in the tumor was increased compared to that in untreated mice, suggesting that ICB treatment might increase cell mobilization and migration from the TdLN to the TME (Figure 1H). Notably, a greater portion of the PA-GFP+ CD8+ T cells infiltrating the tumors of ICB treated mice expressed PD-1, which is a correlate of antigen experience in T cells, compared to untreated mice, further suggesting that ICB treatment can enhance the mobilization of antigen-experienced CD8+ T cells from the TdLN to the TME (Figure 1I).

**Figure 1:**
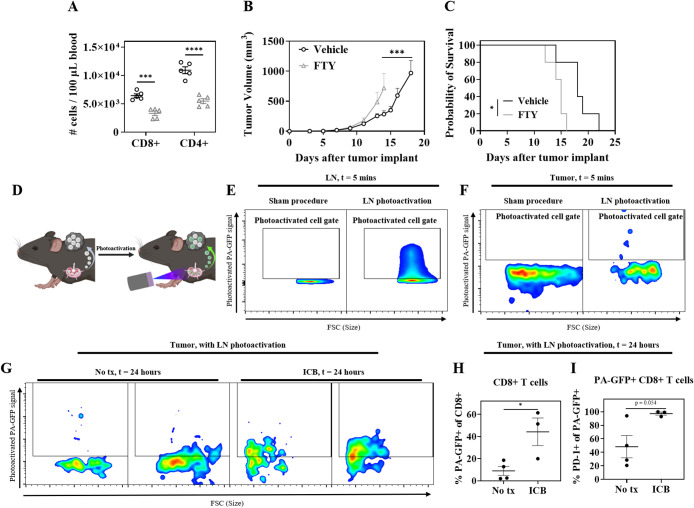
Cell mobilization from TdLNs mediates tumor control and is enhanced by immune checkpoint blockade (ICB). (A–C) B16F10 bearing mice received either 25 μg of FTY-720 or vehicle control i.p. 7 and 9 days after tumor implant. (A) CD8+ and CD4+ T cells in 100 μL of peripheral blood drawn 1 day after beginning treatment. (B) Tumor growth curves and (C) survival curves. (D) Schematic illustrating LN photoactivation in PA-GFP mice. (E) Representative flow cytometry plots of photoactivated PA-GFP signal in lymphocytes from a sham LN (*left*) or a photoactivated LN (*right*) 5 min after photoactivation. (F) Representative flow cytometry plots of photoactivated PA-GFP signal of intratumoral CD8+ T cells after sham procedure (*left*) or TdLN photoactivation (*right*) 5 min after photoactivation. (G) Representative flow cytometry plots of photoactivated PA-GFP signal of intratumoral CD8+ T cells 24 h after TdLN photoactivation with either no treatment (No tx, *t* = 24 h, *left*), or 150 μg each i.p. aPD1 and aCTLA-4 (ICB, *t* = 24 h, *right*). (H) % photoactivated (PA-GFP+) of CD8+ T cells quantified from the data represented in (G). (I) % PD-1+ of PA-GFP+ CD8+ T cells in (H). Data are presented as mean ± standard error of the mean (SEM). In (A–C), *N* = 5; in (E–I), *N* = 3–4. Two-tailed unpaired *t* tests for (A, H, I). Two-way ANOVA with Sidak’s test for (B). Mantel-Cox log rank test for (C). *p < 0.05, **p < 0.01, ***p < 0.001, ****p < 0.0001.

### Preparation and Characterization of the CpG/BPEI-SS/F127-*g*-Gelatin Hydrogel

Locoregionally administered nanocarriers offer advantages for LN drug delivery in the form of their capacity to resist clearance from the site of injection and, when of an appropriate size and charge, to enable lymphatic uptake.^39–43^ Hydrogels, on the other hand, by sustaining drug release over extended periods of time can enable dose sparing to reduce toxicity, potentially improving patient compliance.^27,44,45^ In light of the importance of the TdLN as a source of immunotherapy-responsive T cells for tumor control (Figure 1), we designed a drug delivery system for immunotherapeutic oligonucleotide delivery to the TdLN engineered to simultaneously exploit the advantages each offered by nanocarriers and hydrogels.

Inspired by nonviral gene delivery systems that utilize electrostatic complexation between negatively charged nucleotides and positively charged polymers to form polyplexes, a disulfide-cross-linked polyethylenimine polymer (BPEI-SS-) was prepared as a carrier for CpG oligonucleotides by thiolating and then oxidizing small molecular weight branched PEI (BPEI, 600 Da) (Figure S2).^32–34,46,47^ When characterized using ^1^H NMR, the characteristic peaks of methyl groups from propylene sulfide indicated the thiolation of BPEI (BPEI-SH) through ring opening conjugation of propylene sulfide to amine groups, while peak-shifts and -broadening of ethyl protons adjacent to primary, secondary, and tertiary amines of BPEI demonstrated the successful cross-linking of thiolated BPEI (Figure S3).^33,34^ The interactions of the CpG oligonucleotide with the resultant BPEI-SS- were then characterized.

The mixture of negatively charged CpG (−5.8 mV) and positively charged BPEI-SS- (+16.8 mV) resulted in a material with a positive surface charge (+12.8 mV) (Figures 2A and S4). Using FITC-labeled CpG (CpG-FITC), a red-shift of the absorption spectrum and quenching of the FITC fluorescence were observed for CpG-FITC mixed with BPEI-SS-, suggesting self-quenching of the FITC signal due to the CpG-FITC and BPEI-SS- being in close proximity (Figures 2B,C and S5).^48,49^ Dynamic light scattering (DLS) confirmed that when BPEI-SS- and CpG-FITC were mixed, polyplexes (CpG-FITC/BPEI-SS) formed ranging in size from 500 to 1000 nm depending on the BPEI-SS/CpG mixing weight ratio (Figure 2D). The ability of BPEI-SS- to form polyplexes with CpG (CpG/BPEI-SS) was retained in the absence of FITC, with an average polyplex size of 357.7 nm (Figure 2E). The unusually large size of the polyplex can be ascribed to the intrinsic characteristics of short oligonucleotides such as CpG, which exhibit low charge density and structural rigidity, thereby resulting in loose and less compact complexation with the cationic polymer BPEI-SS-.^50,51^ Accordingly, further size- and surface charge-optimization of CpG/BPEI-SS- polyplex were required to achieve efficient delivery of CpG into the LNs, as engineering lymphatic entry and drainage necessitates smaller nanoparticles (10–200 nm) with slightly negative to neutral charges.^39–43,52–54^

**Figure 2:**
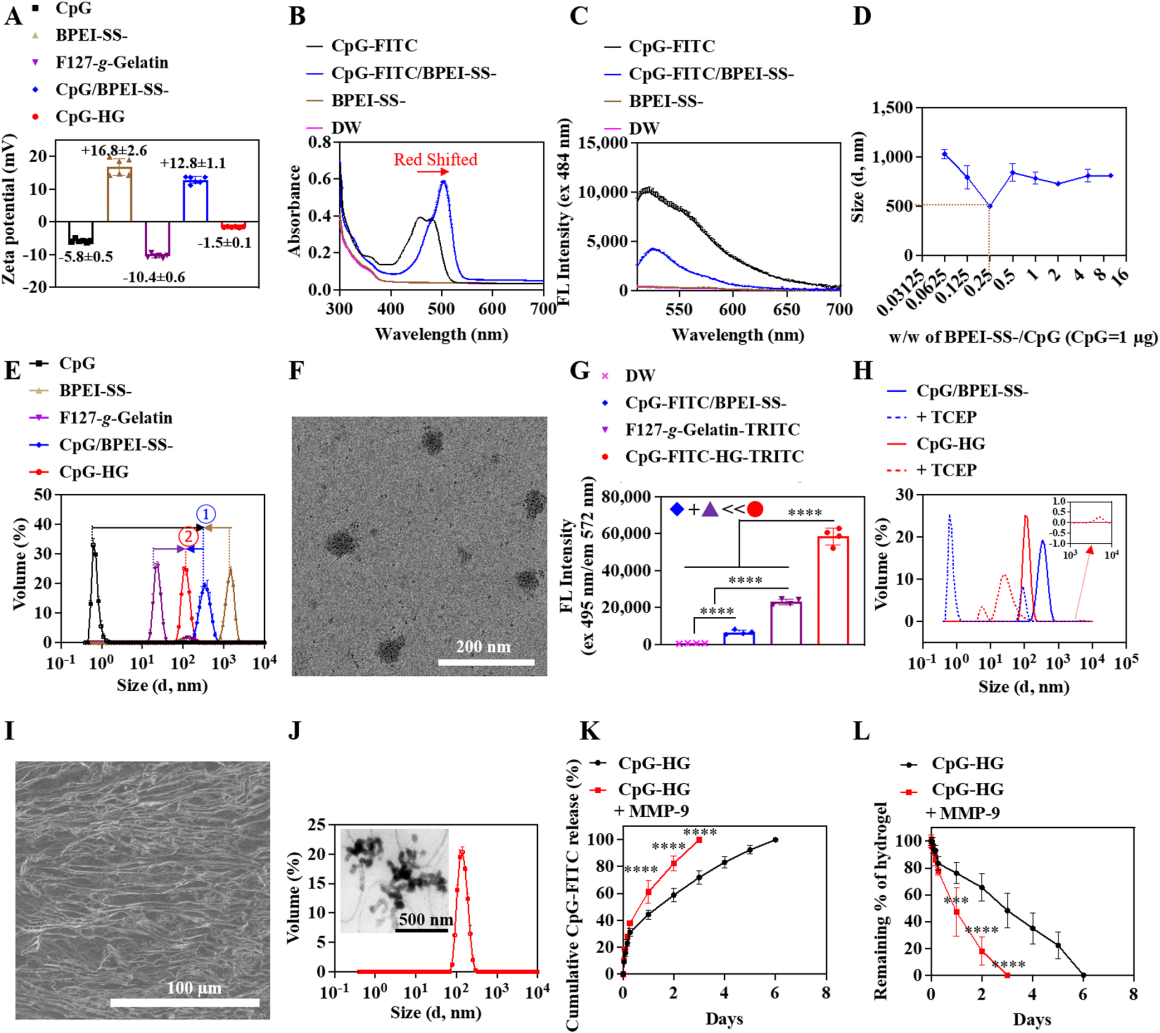
Characterization of CpG-HG polyplex micelles released from the F127-*g*-Gelatin hydrogels. (A) ζ-Potential of free CpG, BPEI-SS-, F127-*g*-Gelatin micelles, CpG/BPEI-SS- polyplexes, and CpG-HG polyplex micelles. (B) Absorbance spectra of CpG-FITC, BPEI-SS-, and CpG-FITC/BPEI-SS- polyplexes. (C) Fluorescence spectra of CpG-FITC, BPEI-SS-, and CpG-FITC/BPEI-SS- polyplexes with 484 nm excitation. (D) DLS measurements of CpG-FITC/BPEI-SS- polyplexes at different weight ratios of BPEI-SS- to CpG-FITC. (E) DLS measurements of free CpG, BPEI-SS-, F127-*g*-Gelatin micelles, CpG/BPEI-SS polyplexes, and CpG-HG polyplex micelles. (F) Representative TEM image of CpG-HG polyplex micelles. (G) Fluorescence resonance energy transfer (FRET) assay of CpG-FITC-HG-TRITC at 495 nm excitation and 572 nm emission. (H) DLS sizes of CpG/BPEI-SS- and CpG-HG exposed to 10 mM TCEP. (I) Representative scanning electron microscopy (SEM) image of F127-*g*-Gelatin hydrogel (4.5 wt %). (J) DLS measurements and a representative transmission electron microscopy (TEM) image of CpG-HG polyplex micelles *in situ* released from F127-*g*-Gelatin hydrogels loading CpG/BPEI-SS- polyplexes. (K) *In vitro* cumulative release of CpG-FITC from CpG-HG hydrogels. (L) *In vitro* stability of CpG-HG hydrogels. Data are presented as mean ± standard deviation (SD). In (A), *N* = 6; in (B, C, J–L), *N* = 3; in (D–H), *N* = 4. ****p < 0.0001, ***p < 0.001, **p < 0.01, *p < 0.05. One-way ANOVA using Tukey’s test for (G). Two-way ANOVA using Tukey’s test for (K, L).

We previously reported a F127-grafted gelatin (F127-*g*-Gelatin) polymer that forms thermosensitive hydrogels to release micelles *in situ*.^35^ Since F127-*g*-Gelatin micelles exhibit a negative charge, we hypothesized that F127-*g*-Gelatin would electrostatically interact with positively charged CpG/BPEI-SS- polyplexes to shrink the size of the polyplexes, followed by self-assembly into polyplex micelles. F127-*g*-Gelatin was synthesized by reacting amines of gelatin with Pluronic F127 (molecular weight 12,600 Da), on which hydroxyl groups were converted to 4-nitrophenyl carbamate in advance (Figure S6). Successful synthesis of F127-*g*-Gelatin was confirmed by assessing the copresence of the characteristic peaks of gelatin and Pluronic F127 in ^1^H NMR after dialysis against deionized water (Figure S7). Quantitative ^1^H NMR revealed that the resultant F127-*g*-Gelatin was composed of ∼62% F127 by mass. As expected, incorporation into negatively charged F127-*g*-Gelatin shrank CpG/BPEI-SS-, resulting in the formation of polyplex micelles with an average diameter of 119.0 nm, a size between that of F127-*g*-Gelatin micelles (43.8 nm) and CpG/BPEI-SS- polyplex (357.7 nm) (Figure 2E). Transmission electron microscopy (TEM) images likewise confirmed the formation of nanoscale polyplex micelles (Figure 2F). Fluorescence resonance energy transfer (FRET) between TRITC-labeled F127-*g*-Gelatin (F127-*g*-Gelatin-TRITC) and CpG-FITC was used to further verify interactions between F127-*g*-Gelatin and CpG/BPEI-SS- polyplexes and CpG-HG polyplex micelle formation. As expected, the CpG-FITC/BPEI-SS- polyplexes showed negligible fluorescence when excited at 547 nm (TRITC excitation), and the F127-*g*-Gelatin-TRITC micelles showed negligible fluorescence when excited at 495 nm (FITC excitation) (Figure S8A,B). The formation of CpG-FITC-HG-TRITC polyplex micelles also did not induce any fluorescence changes in TRITC, showing fluorescence intensity similar to that of F127-*g*-Gelatin-TRITC when excited at 547 nm (Figure S8A). However, the FITC fluorescence intensity was significantly reduced in CpG-FITC-HG-TRITC polyplex micelles when excited at 495 nm compared to that seen with CpG-FITC/BPEI-SS- polyplex (Figure S8B). At the same time, the TRITC fluorescence intensity at 495 nm excitation for CpG-FITC-HG-TRITC polyplex micelles was significantly higher than the sum of TRITC fluorescence intensities for the CpG-FITC/BPEI-SS- polyplex and F127-*g*-Gelatin-TRITC individually at 495 nm excitation (Figure 2G). This revealed CpG-FITC and F127-*g*-Gelatin-TRITC in the mixture to be in close enough proximity to induce FRET, validating the formation of CpG-HG polyplex micelles. It was hypothesized that CpG-HG polyplex micelles would be disrupted under intracellular redox conditions because cleavage of the disulfide bonds could decrease the electrostatic interactions between components below the point necessary for polyplex formation.^32^ Indeed, when CpG-HG polyplex micelles were exposed to simulated intracellular redox conditions (10 mM TCEP), large aggregates and small fragments were observed, indicating that the CpG-HG polyplex micelles would likely undergo disruption in the intracellular environment to release CpG into the cytoplasm (Figure 2H).

To evaluate the sustained release of CpG from the engineered hydrogel *in vitro*, CpG/BPEI-SS- was loaded into the F127-*g*-Gelatin hydrogels by mixing overnight at room temperature, gel formation was confirmed using the vial tilting method at 37 °C, and CpG-HG polyplex micelle release *in situ* after hydrogel formation was evaluated (Figure 2I).^35^ DLS analysis of supernatants released from CpG-HG hydrogels (4.5 wt %) formed at 37 °C revealed the presence of nanoparticles which were of a similar size and ζ-potential to CpG-HG polyplex micelles prepared separately (Figures 2J and S9). This indicates that the simple mixing of CpG/BPEI-SS- polyplexes into F127-*g*-Gelatin thermosensitive hydrogels enabled the release of CpG-HG polyplex micelles without any additional material preparation or modification. Following hydrogel formation, the sustained release of CpG-HG polyplex micelles from the hydrogel formulation was observed (Figure 2K). This sustained release behavior is likely due to the hydrogel decreasing solvent diffusion.^35,55^ In addition to erosion and hydrolysis contributing to hydrogel degradation, CpG-HG polyplex micelle release may be influenced by the presence of matrix metalloproteinases (MMP) constitutively expressed by the epithelium at the injection site.^35,56–58^ To explore the potential for this, the release of CpG from CpG-HG hydrogels in the presence of MMP was evaluated. CpG release was hastened by the presence of MMP-9 and its release directly correlated with hydrogel degradation, as expected due to the interactions of CpG and F127-*g*-Gelatin polymers via BPEI-SS- shown previously (Figures 2K,L and S10). Overall, these results demonstrate that F127-*g*-Gelatin hydrogels can load CpG/BPEI-SS- polyplexes via simple mixing and form thermosensitive hydrogels that release CpG-HG polyplex micelles *in situ* in a sustained manner controlled by gel degradation.

### *In Vivo* Biodistribution and Immunomodulation from CpG Delivered Using Polyplex Micelle-Releasing Hydrogels

Having established the capability of CpG-HG hydrogels to enable the sustained release of CpG *in vitro*, the capacity of CpG-HG to deliver CpG to TdLNs and engineer local immunomodulation was investigated (Figure S11). When administered in the skin (intradermal, i.d.) of the ipsilateral (i.l.) forelimb of B16F10 tumor-bearing mice, CpG-HG exhibited sustained CpG release over 7 days from the site of injection, whereas dose-matched free CpG was rapidly cleared (Figures 3A and S1).

**Figure 3:**
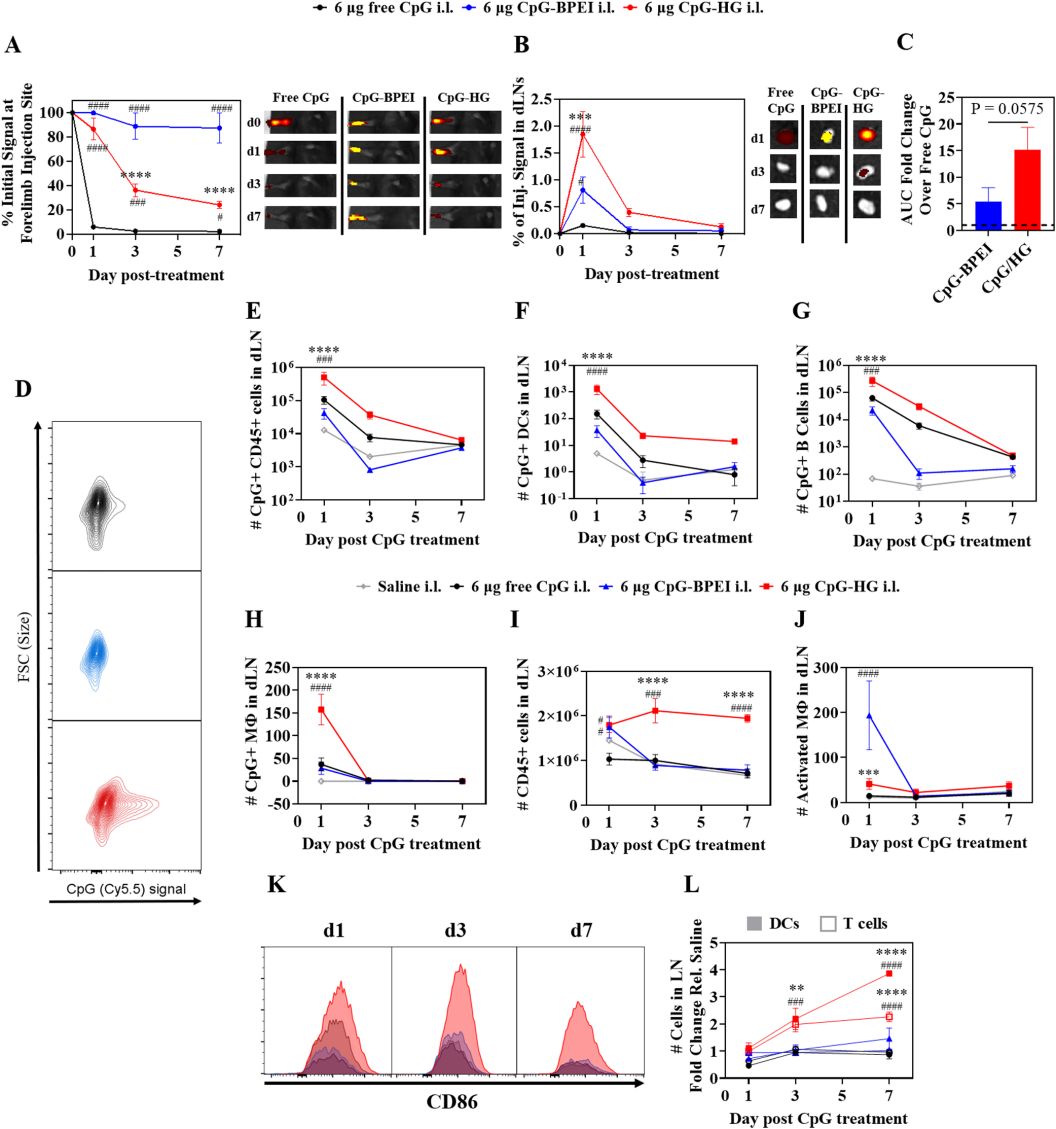
CpG-HG sustains CpG delivery to the draining lymph node (dLN) and adjuvants dLN APCs. Mice received 6 μg of CpG in either free, CpG-BPEI, or CpG-HG form in the i.l. forelimb skin and tissues were collected for IVIS and flow cytometry analysis 1 day, 3 days, and 7 days after CpG treatment. (A, B) Quantification (as a % of initial signal at the injection site) of Cy5.5-labeled CpG using IVIS at (A) the injection site and (B) dLN over time. (C) Area under the curve (AUC) of (B) relative to free CpG. (D) Representative flow cytometry plots of CpG signal in CD45+ cells in dLNs 1 day after CpG administration. (E–H) # of CpG+ (E) CD45+ cells, (F) DCs, (G) B cells, and (H) macrophage (MΦ) in dLNs over time after CpG administration. (I, J) # of (I) CD45+ cells and (J) activated MΦ in dLNs over time after CpG administration. (K) Representative flow cytometry histograms of CD86 (MFI) on DCs in the dLN 1, 3, and 7 d after CpG administration. (L) Fold change over time relative to saline in the # of T cells and DCs in the dLN. *N* = 5. Data are presented as mean ± SEM. ####*p* < 0.0001, ###*p* < 0.001, ##*p* < 0.01, #*p* < 0.05 compared to free CpG. ****p < 0.0001, ***p < 0.001, **p < 0.01, *p < 0.05 compared to CpG-BPEI. Two-tailed unpaired *t* test for (C). Two-way ANOVA using Tukey’s test for (A, B, E–J, L).

When delivered in CpG/BPEI-SS- polyplexes (hereafter CpG-BPEI), CpG levels at the injection site were maintained over 7 days, presumably due to the polyplex’s positive surface charge and size much larger than ECM pores resulting in comparatively limited transport through the tissue interstitium. When evaluating LNs draining the local injection site (dLN), delivery from CpG-HG resulted in overall higher CpG accumulation relative to free CpG and CpG-BPEI over 1 week, a result that can be attributed to the released micelles’ slightly negative surface charge and hydrodynamic size being amenable to efficient lymphatic delivery (Figure 3B). Interestingly, while exhibiting less LN accumulation compared to hydrogel-delivered CpG, CpG delivered from BPEI polyplexes showed higher LN accumulation than free CpG 1 day after administration, an effect that, in light of the CpG-BPEI’s retention at the injection site, may be due to the migration via the lymphatics of APCs from the injection site.^39^ CpG accumulation in tissues outside of the skin injection site and dLN in CpG-HG treated mice, such as the tumor, spleen, and the nondraining (contralateral) LNs (NdLN), was limited, particularly relative to CpG-BPEI (Figure S12A–C). CpG-HG thus facilitates the efficient and prolonged accumulation of CpG in LNs draining its injection site with minimal exposure in systemic off-target tissues.

When evaluating immune modulatory effects of CpG treatment, mice treated with CpG-HG showed significantly higher numbers of CpG+ CD45+ lymphocytes in dLNs over time compared with mice treated with free CpG and CpG-BPEI (Figure 3D,E). Notably, CpG-HG enabled significantly greater CpG delivery to LN APCs, including DCs and B cells, than free CpG or CpG-BPEI (Figure 3F,G). Interestingly, CpG-BPEI exhibited significantly lower CpG delivery to LN DCs and B cells relative not only to CpG-HG but also to free CpG despite demonstrating overall higher levels of dLN accumulation compared to free CpG. This is presumably due to free CpG and CpG-BPEI accessing the dLN via different mechanisms, namely direct lymphatic drainage vs migratory APCs respectively, due to the divergent hydrodynamic sizes of free CpG and CpG-BPEI. In contrast to the trends seen for DCs and B cells, the number of CpG+ MΦ in CpG-HG treated dLNs was significantly increased vs free CpG and CpG-BPEI at 1 day after treatment but had returned to baseline by 3 days after treatment (Figure 3H). Similarly, CpG+ CD8+ and CD4+ T cells were modestly increased in CpG-HG treated dLNs only at early time points after treatment and subsequently decreased to levels similar to those observed in free CpG and CpG-BPEI dLNs (Figure S13A,B). Overall, these results indicate that CpG-HG substantially improves LN accumulation of CpG by sustained release of CpG into the lymphatics, resulting in enhanced uptake of CpG by local APCs.

The immunomodulatory effects resulting from increased and sustained delivery of CpG to LN-resident APCs on dLN lymphocytes were also evaluated. Both hydrogel- and BPEI-delivered CpG led to significantly greater expansion of CD45+ cells than free CpG in dLNs 1 day after treatment. However, while the number of CD45+ cells in dLNs treated with CpG-BPEI decreased to a level similar to those treated with free CpG by 3 days after treatment, CpG-HG treated dLNs maintained CD45+ cell expansion over 7 days (Figure 3I). Interestingly, while CpG-HG treated dLNs exhibited increased CpG uptake by MΦ 1 day after treatment, MΦ activation did not correspond with CpG uptake: only CpG-BPEI treated dLNs exhibited significant MΦ activation 1 day after treatment (Figure 3H,J). In contrast, CpG-HG not only increased the total number of DCs but also increased DC activation, as measured by CD86 expression, up to 7 days after treatment, while free CpG and CpG-BPEI treated dLNs saw minimal DC expansion and activation (Figure 3K,L). This pattern extended to DC subsets as well, with CpG-HG treated dLNs showing increased numbers of both total and activated type 1 conventional DCs (cDC1s) and type 2 conventional DCs (cDC2s) in dLNs after 7 days (Figure S14A–F). A median of 45% of activated DCs in CpG-HG treated dLNs had detectable CpG uptake 1 day after treatment compared to a median of 11.3 and 3.2% in free CpG and CpG-BPEI treated dLNs respectively, suggesting that the increased DC activation in CpG-HG treated dLNs is likely primarily a result of CpG uptake (Figure S14G). CpG-HG also led to prolonged increases in total B cell numbers in dLNs and activated B cells compared to free CpG and CpG-BPEI, illustrating the capability of CpG-HG to achieve enhanced delivery of CpG to multiple types of dLN APC (Figure S14H,I). cDC1s and cDC2s in the dLN play critical roles in priming CD8+ T cells and CD4+ T cells, respectively.^59–61^ As a result of CpG delivery to dLN cDC1s and cDC2s, increased numbers of both CD8+ and CD4+ T cells were observed in CpG-HG treated dLNs compared to free CpG or CpG-BPEI treated dLNs, with CpG-HG treated dLNs seeing a longer period of increased T cell presence (7 days) compared to free CpG or CpG-BPEI treated dLNs (<3 days) (Figures 3L and S14J,K). These immunomodulatory effects were primarily restricted to the dLN, with no significant cell expansion being observed in the spleen after CpG-HG administration compared with free CpG or CpG-BPEI (Figure S15A–E). While DCs and T cells were modestly increased in NdLNs at 1–3 days and 7 days after CpG-HG administration respectively, this increase was quantitatively less than observed in the dLNs and no expansion of total CD45+ cells or B cells was observed in NdLNs (Figure S15F–J). Taken together, these results indicate that CpG-HG enables enhanced and sustained CpG uptake by dLN APCs, augmenting and prolonging the local adjuvant effects of CpG to achieve sustained DC activation and sustained DC and T cell expansion in dLNs.

### CpG Formulation Results in Distinct Patterns of CpG Delivery to dLN APCs

The mechanism by which CpG-HG delivers CpG to dLN APCs was next evaluated, in particular: whether CpG-HG primarily delivers CpG-containing micelles or sustains the release of free CpG into the lymphatics *in vivo*, whether different APC subsets access micelles to different extents, and whether delivery of CpG from CpG-HG influences the intracellular spatial distribution of CpG. To investigate these questions, fluorescently labeled free CpG, CpG-BPEI, or CpG-HG (CpG and HG labeled with Cy5.5 and FITC, respectively) were administered i.d. in the i.l. forelimb of B16F10 tumor-bearing mice. One day after CpG administration, dLNs were collected and cells were analyzed using imaging flow cytometry (Figure 4A). Within the CpG-HG group, some CpG+ cells showed clear intracellular copresence of CpG (*red*) and HG (*green*) fluorescent signals, indicating a degree of CpG and F127-*g*-Gelatin codelivery, and demonstrating that CpG-HG releases intact CpG-containing polyplex micelles *in vivo* rather than simply sustaining the release of free CpG at the injection site (Figure 4B). To investigate to what extent the CpG formulation influenced the intracellular spatial distribution of CpG after uptake, two measurements were calculated for CpG+ cells: the distance between CpG fluorescence and the cell centroid, and the area of the cell with CpG fluorescence (as a measure of intracellular CpG dispersion). CpG+ B cells in CpG-BPEI treated dLNs were found to have CpG more distally located compared to those in free CpG treated dLNs, while CpG+ B cells in CpG-HG treated dLNs trended similarly to CpG-BPEI (Figure 4C). CpG+ DCs, by contrast, exhibited no conclusive difference in distal vs central CpG localization by formulation (Figure 4D). Similarly, when comparing the average intracellular area occupied by CpG, CpG within B cells in free CpG and CpG-HG treated dLNs was distributed across a significantly greater intracellular area than in CpG-BPEI treated dLNs while DCs again showed no difference in CpG distribution between formulations (Figure 4E,F). These data suggest that the CpG formulation exerts distinct effects on CpG localization within B cells but not DCs.

**Figure 4:**
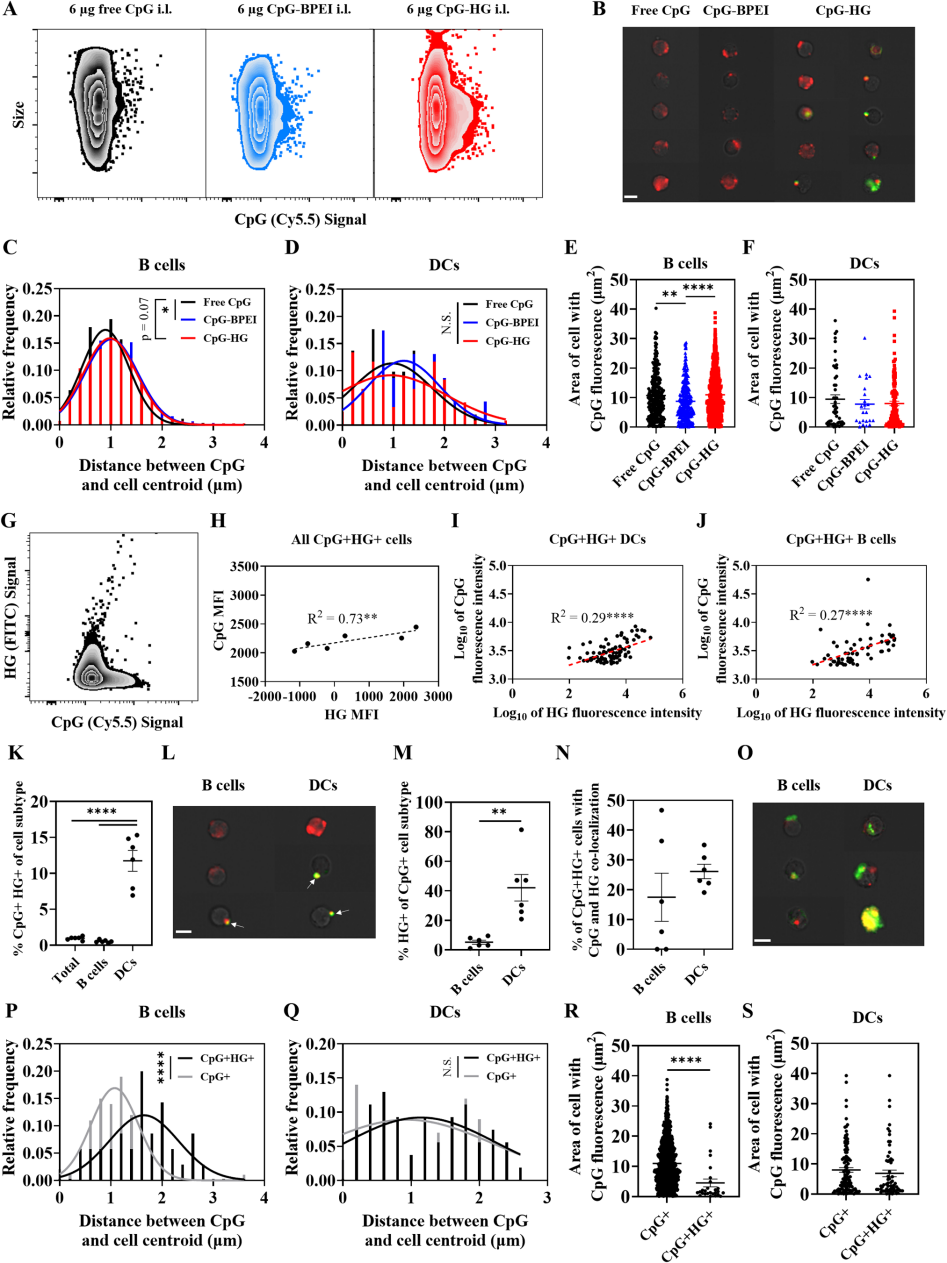
CpG-HG shows distinct patterns of delivery to DCs and B cells in the dLN. Mice received 6 μg of CpG in either free, CpG-BPEI, or CpG-HG form in the i.l. forelimb skin and LNs were collected for imaging flow cytometry (IFC) analysis 1 day after treatment. (A) Representative IFC plots of CpG+ cells in dLNs. (B) Representative IFC images of CpG+ cells in dLNs from (A). Plots (C–F) are calculated from the images represented in (B). (C, D) Histograms illustrating distance between intracellular CpG and the cell centroid for individual CpG+ (C) B cells and (D) DCs. (E, F) Intracellular area occupied by CpG for individual CpG+ (E) B cells and (F) DCs. (G) Representative IFC plot showing CpG+HG+ cells in CpG-HG treated dLNs. Plots (H–K, M) are calculated from the data represented in (G). (H) Correlation between CpG MFI and HG MFI of all CpG+HG+ cells. (I, J) Correlation between CpG fluorescence intensity and HG fluorescence intensity for individual CpG+HG+ (I) DCs and (J) B cells. (K) Comparison of % of either all cells, B cells, or DCs in CpG-HG treated dLNs that are CpG+HG+. (L) Representative IFC images from CpG+ B cells and DCs in CpG-HG treated dLNs; arrows indicate micelle presence. (M) % of cells that are also HG+ of CpG+ B cells and DCs in CpG-HG treated dLNs. (N, O) Of CpG+HG+ B cells and DCs, (N) the % of cells that exhibit colocalization of CpG and HG signals and (O) representative IFC images of CpG+HG+ B cells and DCs used to calculate (N). Plots (P–S) are calculated from the images represented in (O). (P, Q) Histograms illustrating distance between intracellular CpG and the cell centroid for individual CpG+ cells compared to individual CpG+HG+ cells in CpG-HG treated dLNs for (P) B cells and (Q) DCs. (R, S) Comparison of intracellular area occupied by CpG for individual CpG+ cells vs individual CpG+HG+ cells in CpG-HG treated dLNs for (R) B cells and (S) DCs. *N* = 3–6 biological replicates for (H, K, M, N). Data are presented as mean ± SEM. ****p < 0.0001, ***p < 0.001, **p < 0.01, *p < 0.05. One-way ANOVA using Tukey’s test for (C–F, K). Two-tailed unpaired *t* tests for (M, N, P–S). Dashed lines indicate linear regression trendlines in (H–J). Scale bars in (B, L, O) represent 7 μm.

To gain further insight into CpG delivery from CpG-HG, cells in the CpG-HG group with the detectable presence of both CpG and HG signals (CpG+HG+) were analyzed (Figure 4G). Within all CpG+HG+ cells, there was a correlation between the CpG median fluorescence intensity (MFI) and the HG MFI, suggesting a linear relationship between CpG uptake and micelle uptake in this cell population (Figure 4H). A similar trend, though weaker, was seen when analyzing individual CpG+HG+ DCs and B cells (Figure 4I,J). Interestingly, CpG+HG+ cells were overwhelmingly DCs rather than B cells despite B cells having some detectable CpG and HG signal copresence, indicating that while both DCs and B cells access intact CpG-containing polyplex micelles B cells do so to a comparatively limited extent and further suggesting distinct patterns of micelle access to DCs vs B cells (Figure 4K–M). Despite this, there was no significant difference in CpG and HG signal colocalization as a % of CpG+HG+ DCs vs CpG+HG+ B cells (Figure 4N,O). Interestingly, when comparing the spatial distribution of CpG within CpG+HG+ cells vs all CpG+ cells in CpG-HG treated dLNs (Figure 4P–S), CpG+HG+ B cells were found to have both less dispersed and more distally located CpG than CpG+ B cells (Figure 4P,R). This suggests that while CpG+HG+ B cells do access intact CpG-containing polyplexmicelles, most CpG+ B cells access CpG in a micelle-independent manner. The limited micelle uptake visible in B cells despite the micelle diameter (∼100 nm) being too large to enter the LN parenchyma via reticular conduits suggests some degree of paracellular transport of micelles across the lymphatic endothelial cells forming the subcapsular sinus (SCS) floor.^62–64^ However, the fact that so few B cells have CpG and HG signal copresence suggests that this mechanism of transport is relatively minor and that B cells in CpG-HG treated dLNs primarily access CpG apart from micelles. Nanoparticles with polyethylene glycol (PEG) coronas are known to be capable of complement-dependent transcytosis into LNs via subcapsular sinus MΦ.^65,66^ Because the CpG-containing micelles are disrupted in intracellular redox conditions and in light of CpG-containing micelle uptake by MΦ not resulting in MΦ activation (Figure 3), it is possible that subcapsular sinus MΦ mediates transcytosis of CpG-containing micelles but that the micelle structure is degraded during the process, resulting in the release of micelle-free CpG into the follicular zone of the dLN and subsequent uptake of released CpG by B cells. CpG+HG+ DCs, on the other hand, were found to display similarly dispersed and distally located CpG as CpG+ DCs (Figure 4Q,S). In contrast to B cells, this suggests that most CpG+ DCs in CpG-HG treated dLNs access CpG in a micelle-dependent manner—potentially due to lymphatic sinus-associated DCs taking up CpG-containing polyplex micelles while sampling subcapsular sinus lymph.^67,68^ Taken together, these results show that CpG-HG releases intact, lymph-draining, CpG-containing micelles *in vivo* that access dLN DCs and B cells by distinct mechanisms and that CpG formulations dictate distinct patterns of intracellular CpG localization.

### *In Vivo* Therapeutic Effects of CpG-HG Hydrogels

In light of the potent immunomodulatory effects observed in the dLN in response to CpG-HG treatment and our previous work exploring the therapeutic benefit of daily adjuvant delivery to TdLNs, we hypothesized that CpG-HG sustaining CpG delivery to LNs from a single injection might exert more potent antitumor effects *in vivo* as compared to a single treatment of unformulated CpG.^14^ When B16F10 tumor-bearing mice were treated with saline, F127-*g*-Gelatin (hereafter blank HG), free CpG, or CpG-HG in the i.l. forelimb 4 days after tumor implant, CpG-HG treatment enabled a degree of tumor control relative to free CpG and blank HG treatments and was well-tolerated as indicated by mouse body weight, though it did not significantly improve survival (Figure 5A–C). However, when B16F10 tumor-bearing mice were treated with either saline, blank HG, free CpG, or CpG-HG in the i.l. forelimb 4 days after tumor implant, followed by i.p. ICB or isotype antibodies 5, 7, 9, 11, 13, 15, and 18 days after tumor implantation, the combination of CpG-HG and ICB enabled both improved tumor control and improved survival while still being well tolerated (Figure 5D–F).

**Figure 5:**
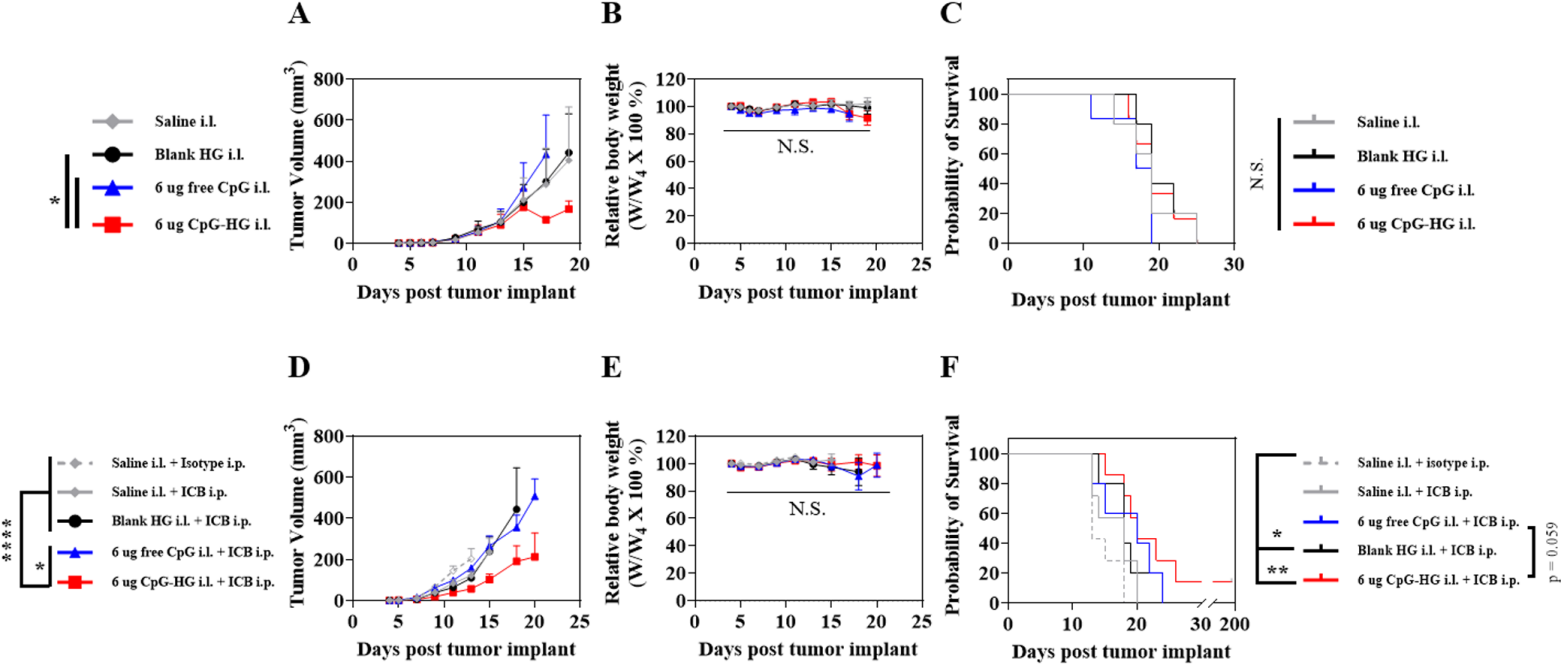
CpG-HG treatment improves tumor control and synergizes with systemic ICB to improve survival. (A–C) B16F10-bearing mice were treated with either saline, blank HG, 6 μg of free CpG, or 6 μg of CpG-HG in the i.l. forelimb skin 4 days after tumor implant. (A) Tumor growth curves, (B) body weight relative to start of treatment, and (C) survival. (D–F) B16F10-bearing mice were treated with saline, blank HG, 6 μg of free CpG, or 6 μg of CpG-HG in the i.l. forelimb skin 4 days after tumor implant followed by either 150 μg each i.p. aPD-1 + aCTLA-4 (ICB) or isotype antibodies 5, 7, 9, 11, 13, 15, and 18 days after tumor implant. (D) Tumor growth curves, (E) body weight relative to start of treatment, and (F) survival. *N* = 5–7. Data are presented as mean ± SEM. ****p < 0.0001, ***p < 0.001, **p < 0.01, *p < 0.05. Two-way ANOVA using Tukey’s test for (A, B, D, E). Mantel-Cox log rank test for (C, F).

Interestingly, when peripheral blood was collected from mice treated with either saline, blank HG, free CpG, or CpG-HG but no isotype or ICB treatment and analyzed for cells that might have egressed from the adjuvanted dLNs, no significant change in CD8+ T cell mobilization was observed up to a week after treatment including in subsets such as PD-1+, stem-like (PD-1+TCF1+Tim3−), effector-like (PD-1+TCF1-Tim3+), or cycling Ki-67+ CD8+ T cells (Figure S16A–E). In light of this, we hypothesized that the synergy observed between CpG-HG and systemic ICB might be due to ICB driving the therapeutic mobilization of expanded T cells from the dLN.

To test this hypothesis, B16F10 tumor-bearing mice received either saline, free CpG, blank HG, or CpG-HG in the i.l. skin 4 days after tumor implant followed by a single administration of either ICB or isotype antibodies i.p. 5 days after tumor implant (Figure S1). Peripheral blood was then collected starting from 2 days after CpG treatment (1 day after ICB treatment) and continuing 3, 5, 7, and 9 days after CpG treatment. Strikingly, a marked increase in PD-1+ CD8+ T cells and cycling Ki-67+ CD8+ T cells in blood circulation was observed only for the combination of CpG-HG and ICB (Figures 6A,B and S17A,B). Notably, PD-1 expression on T cells has been observed to correlate to T cell activation by APCs presenting cognate antigen and to T cell reactivity to tumor antigen, and so can be used to denote antigen-experienced T cells.^11,69–74^ This increase in T cell mobilization for mice treated with both CpG-HG and ICB was even more profound when considering recently proliferated antigen-experienced (PD-1+Ki-67+) CD8+ T cells (Figures 6C and S17C). Not only was the magnitude of mobilization of these cells greatest for mice treated with CpG-HG and ICB, but the duration of the circulating T cell response was also greatest in mice treated with CpG-HG and ICB (Figure 6D). The significance of circulating PD-1+Ki-67+ CD8+ T cells has been previously highlighted both by studies indicating that these cells are representative of a tumor-reactive T cell response to treatment and by research identifying increases in this population following ICB treatment as a potential on-treatment biomarker of ICB response in humans when accounting for pretreatment tumor burden.^75–78^ To examine whether sustained adjuvanting of the dLN with CpG-HG might influence this biomarker of response, the maximum fold change in circulating PD-1+Ki-67+ CD8s was determined for each animal, and the ratio of fold change to pretreatment tumor burden was then calculated for each animal. Classification and regression tree (CART) analysis was then used to separate animals into two groups internally homogeneous for survival based on the fold change to tumor burden ratio (Figure 6E,F). Interestingly, mice treated with both CpG-HG and ICB not only tended to see greater circulating T cell response than mice receiving ICB but not CpG-HG (ICB controls) or mice not receiving ICB (isotype controls) but were also proportionally more likely to be associated with greater-than-median survival (“responders”) than mice in the ICB control or isotype control groups (Figure 6G). To confirm that the observed T cell mobilization observed in response to CpG-HG and ICB treatment did not correlate with treatment-related toxicity, blood plasma from treated mice was analyzed for alanine aminotransferase (ALT) and aspartate aminotransferase (AST) enzyme activity 1 day after ICB treatment with no significant changes in ALT or AST activity observed in response to combination therapy (Figure S18).

**Figure 6:**
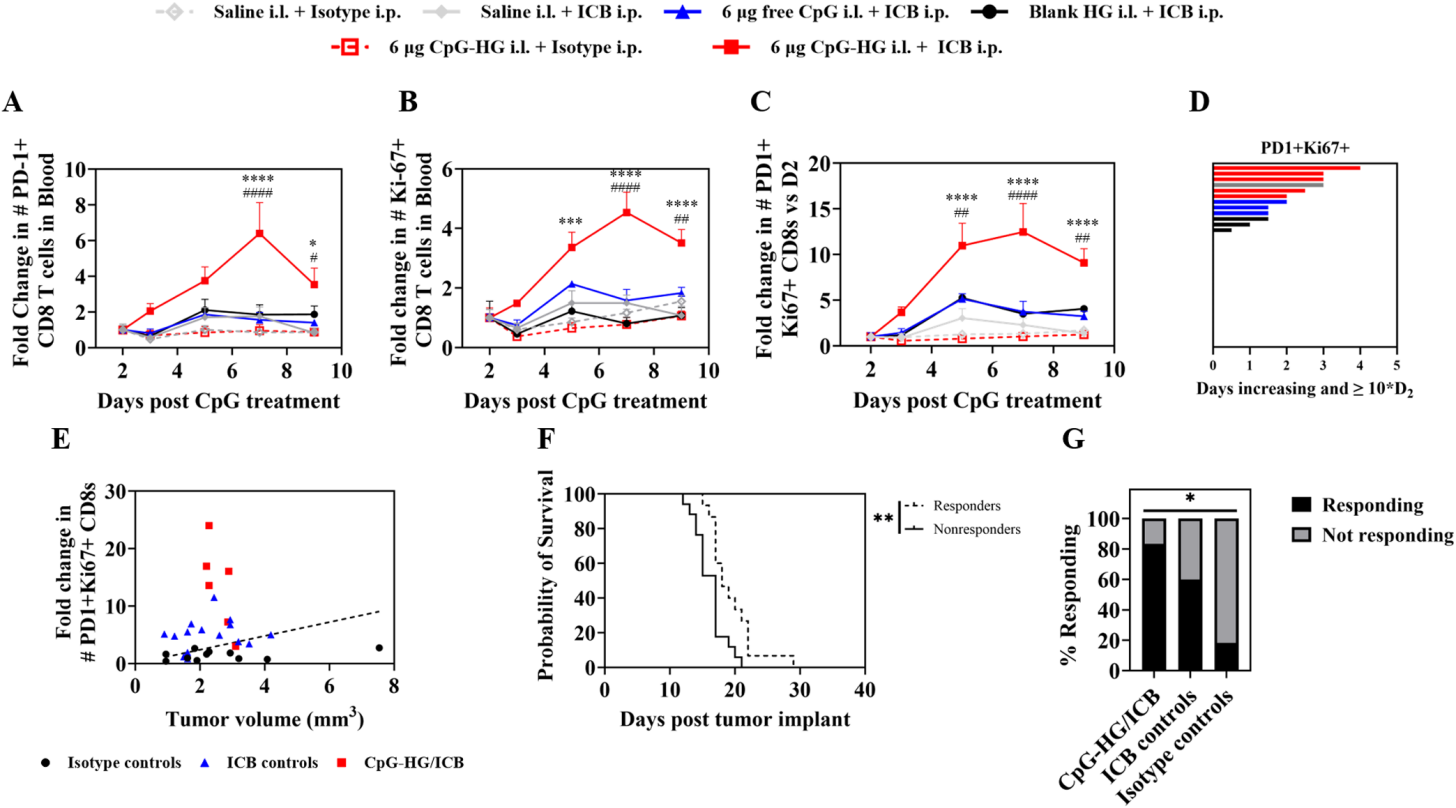
Single administration of ICB following CpG-HG treatment sustains a potent circulating T cell response. B16F10-bearing mice were treated with saline, blank HG, 6 μg of free CpG, or 6 μg of CpG-HG in the i.l. forelimb skin 4 days after tumor implant followed by either 150 μg each i.p. aPD-1 + aCTLA-4 (ICB) or isotype antibodies 5 days after tumor implant. Peripheral blood was collected 2, 3, 5, 7, and 9 days after CpG treatment for flow cytometry analysis. (A–C) Fold change relative to 2 days after CpG treatment in # of (A) PD-1+ CD8+ T cells, (B) Ki-67+ CD8+ T cells, and (C) PD-1+Ki-67+ CD8+ T cells in 100 μL of peripheral blood. (D) Swimmer plot illustrating duration of circulating T cell response as measured by consecutive days over which the # of PD-1+Ki67+ CD8+ T cells in the blood increase. (E) Maximum fold change in # of PD-1+Ki-67+ CD8+ T cells vs pretreatment tumor volume, stratified by survival (dashed line). Animals are pooled for CART analysis based on receiving both CpG-HG and ICB (CpG-HG/ICB), ICB but not CpG-HG (ICB controls), or isotype antibodies (Isotype controls). Data points falling above the dashed line are considered responders; data points falling below the dashed line are considered nonresponders. (F) Survival of responders vs nonresponders from (E). (G) % of mice in group either responding or not responding. *N* = 5–6. Data are presented as mean ± SEM. In (A–C), ****p < 0.0001, ***p < 0.001, **p < 0.01, *p < 0.05 compared to saline + ICB. ####*p* < 0.0001, ###*p* < 0.001, ##*p* < 0.01, #*p* < 0.05 compared to free CpG + ICB. Two-way ANOVA using Tukey’s test for (A–C). Mantel-Cox log rank test for (F). Chi-square test for (G).

In light of the fact that a single administration of ICB was observed to mobilize CD8+ T cells in significant quantities and for a meaningful duration, we hypothesized that CpG-HG may facilitate fewer ICB doses. To investigate this, B16F10 tumor-bearing mice were treated with either saline, free CpG, or CpG-HG 4 d after tumor implant. Following this, mice received either a single treatment of ICB i.p. 5 days after tumor implant or three treatments of ICB i.p. 5 days, 7 days, and 9 days after tumor implant, respectively. As anticipated, mice receiving CpG-HG and three ICB treatments exhibited reduced tumor growth and somewhat improved survival compared to mice receiving either saline or free CpG and three treatments of ICB (Figure 7A,B). Similarly, mice receiving CpG-HG and a single treatment of ICB exhibited greater tumor control and improved survival compared to those receiving saline or free CpG and a single treatment of ICB (Figure 7C,D). Interestingly, however, mice receiving CpG-HG exhibited similar tumor control and survival regardless of whether they received a single treatment or three treatments of ICB, indicating that the systemic antitumor immune response provoked by CpG-HG followed by a single administration of ICB was sufficient to reduce the need for further administrations of ICB while maintaining therapeutic benefit Figure 7E,F).

**Figure 7:**
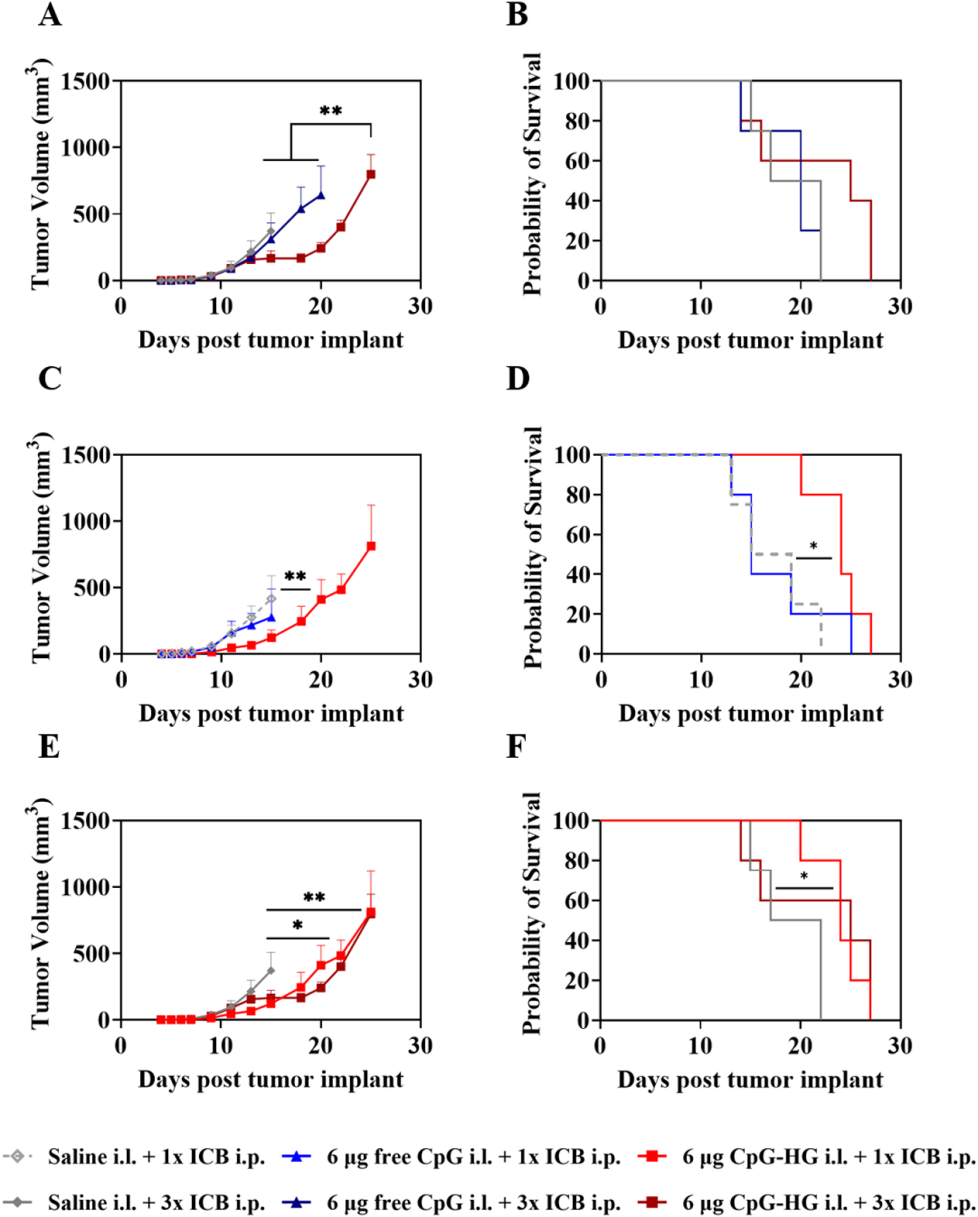
Sustained CpG delivery to TdLNs from CpG-HG enhances the synergies of CpG treatment with ICB with fewer administrations. B16F10-bearing mice were treated with either saline, 6 μg of free CpG, or 6 μg of CpG-HG in the i.l. forelimb skin 4 days after tumor implant followed by either a single dose (1×) i.p. of 150 μg each aPD-1 + aCTLA-4 (ICB) 5 days after tumor implant or three doses (3×) i.p. of ICB 5, 7, and 9 days after tumor implant. (A, B) For mice receiving 3× ICB, (A) tumor growth curves and (B) survival. (C, D) For mice receiving 1× ICB, (C) tumor growth curves and (D) survival. (E, F) Comparison of mice receiving 3× ICB to mice receiving CpG-HG and 1× ICB in (E) tumor growth and (F) survival. *N* = 4–5. Data are presented as mean ± SEM. Two-way ANOVA using Tukey’s test for (A, C, E). Mantel-Cox log rank test for (B, D, F).

## Conclusions

The limited efficacy and significant toxicities associated with ICB therapies have motivated investigations into ways by which efficacy might be improved and dose sparing achieved.^79–81^ Recently, TLR9 agonists such as CpG oligonucleotides which were originally investigated with limited effect as cancer monotherapies have been revisited with the goal of potentiating the effects of ICB therapy.^82,83^ However, despite promising signals in early stage clinical trials, approaches combining TLR9 agonists and ICB (including both PD-1 and CTLA-4 targeted agents) have yet to be successful in late stage clinical trials.^84,85^ Notably, these trials have often utilized either systemic administration, which results in limited drug access to cells of interest, or intratumoral administration, which targets a therapeutically relevant but immunologically hostile tissue. On the other hand, due to their roles as specialized tissues affording localized immune education resulting in systemic immunity, TdLNs have been highlighted as potential immunotherapeutic targets and may hold the key to more effective toll like receptor (TLR)-based immunotherapy but may not be adequately accessed by TLR9 agonist following systemic or even intratumoral administration.^10,17,35^ Here, we developed a thermosensitive, redox-reactive hydrogel system for the sustained delivery of an oligonucleotide adjuvant to dLNs. CpG-HG combines attractive features of both conventional sustained release and conventional nanocarrier systems, exhibiting both prolonged drug release from the site of injection and efficient drug delivery in the form of CpG-containing micelles to the dLN. CpG is efficiently accessed by APCs within the dLN, and CpG-accessing B cells and DCs exhibit different patterns of intracellular CpG localization that are distinct from intracellular localization for free CpG. This sustained delivery of CpG to dLN APCs prolonged DC and T cell expansion within dLNs. When delivered prior to treatment with systemically administered ICB, this expanded T cell pool resulting from sustained CpG delivery to TdLNs strengthened and prolonged systemic circulating T cell immunity, increased therapeutic potency, and obviated the need for repeat dosing. Sustained LN adjuvanting via CpG-HG thus has significant potential to enhance therapeutic responses to ICB and achieve ICB dose sparing.

## Experimental Section

### Materials

BPEI (BeanTown Chemical, 600 Da), methanol, 70 μm strainers (Corning), and collagenase D (Roche) were obtained from VWR Scientific. 37% HCl, propylene sulfide, dimethyl sulfoxide (DMSO), Pluronic F127, dichloromethane (DCM), 4-nitrophenyl chloroformate (*p*-NPC), diethyl ether, gelatin type A (300g bloom), and Red Blood Cell Lysing Buffer Hybri-Max were purchased from Sigma-Aldrich (St. Louis, MO). D_2_O was purchased from Cambridge Isotope Laboratories, Inc. (Andover, NA). Dialysis tubes (MWCO 3.5 and 100 kDa) were obtained from Spectrum Industries (Los Angeles, CA). Phosphate buffered saline (PBS), eBioscience Foxp3/Transcription Factor Staining Buffer Set (Invitrogen), fluorescein isothiocyanate (FITC), tetramethylrhodamine (TRITC), and Ethylenediaminetetraacetic acid (EDTA) were purchased from ThermoFisher Scientific. CpG oligonucleotides (5′-TCC ATG ACG TTC CTG ACG TT-3′) with or without FITC or Cy5.5 at the 5′ end were purchased from Microsynth AG (Balgach, Switzerland). aCTLA-4 (Clone: UC10-4F10-11, Cat#: BP0032), and aPD-1 (Clone: RMP1-14, Cat#: BP0146), polyclonal American Hamster IgG (aCTLA-4 isotype, Cat#: BP0091), and Rat IgG2a (aPD-1 isotype, Clone; 2A3. Cat#: BP0089) were purchased from BioXCell (Lebanon, NH). Vendors for Zombie Aqua fixable viability dye and staining antibodies used in flow cytometry are listed in Supporting Table T1.

### Synthesis of BPEI-SS-

One g portion of BPEI was solubilized in 20 mL of deionized water, with the pH then adjusted to 7.5 using 2 N HCl. The neutralized BPEI solution was lyophilized and then solubilized into 30 mL of methanol. 640 mg of propylene sulfide (5 equiv to BPEI) was added to BPEI solution, and the reaction allowed to proceed with vigorous stirring at 60 °C for 2 d. The resultant BPEI-SH was precipitated in 1.5 L of cold diethyl ether and then oxidized in 100 mL of DMSO for 2 d. The solution was dialyzed against deionized water (MWCO = 3.5 kDa) for 2 days and then freeze-dried to yield BPEI-SS-.

### Synthesis of F127-*g*-Gelatin

Twenty g of Pluronic F127 was solubilized in 50 mL of DCM, which was then added dropwise to 3.2 g of *p*-NPC in 50 mL DCM. The volume of the solution was reduced to 30% by using rotary evaporation after overnight reaction with vigorous stirring. *p*-NPC activated F127 was yielded after precipitation in 2750 mL of cold diethyl ether and filtration. *p*-NPC activated F127 was solubilized in 50 mL of ethanol, which was added dropwise to 10 g of Gelatin in 350 mL of deionized water with 1 mL of TEA. The final volume of the mixture was adjusted to be 1 L containing 10 mL of TEA. After overnight reaction, the solution was dialyzed against deionized water (MWCO 100 kDa) for 1.5 days and then freeze-dried to yield F127-*g*-Gelatin.

### Characterization of Polymers

The chemical composition of BPEI-SS- and F127-*g*-Gelatin in D_2_O was analyzed with Bruker Avance 400 MHz FT-NMR using Topspin v3.0 software and MestreNova NMR v11.

### Preparation and Characterization of CpG/BPEI-SS- Polyplex and CpG/BPEI-SS-/F127-*g*-Gelatin (CpG-HG) Polyplex Micelles

CpG (1 μg) and BPEI-SS- were mixed at different weight ratios in PBS, followed by pipetting and incubation for 20 min at room temperature. Absorbance and fluorescence spectral changes of CpG-FITC in the mixture with BPEI-SS- were recorded by using a Synergy H4 microplate reader (BioTek). CpG/BPEI-SS- polyplex (w/w = 0.25, CpG = 1 μg) was mixed with an equal volume of F127-*g*-Gelatin (10 μg μL^–1^) to make CpG-HG polyplex micelles. The size and ζ-potential of materials were determined by using dynamic light scattering (DLS) and Zetasizer Nano ZS (Malvern Instruments). High-resolution FEI Tecnai G2 F30 TEM (FEI Company) was employed to visualize CpG-HG polyplex micelles. Intracellular redox-sensitive behavior of CpG/BPEI-SS-and CpG-HG was evaluated by examining DLS size changes under 10 mM TCEP.

### FRET Assay

TRITC labeled F127-*g*-Gelatin (F127-*g*-Gelatin-TRITC) was prepared by reacting 8 mg of F127-*g*-Gelatin in 1 mL of PBS with 160 μL of 1 mg mL^–1^ TRITC in DMSO at room temperature for 2 h and then purifying samples with Amicon Ultra centrifugal filters (Milipore, MWCO 30 kDa) at 4000 g and 4 °C for 20 min. The fluorescence intensity of CpG-FITC/BPEI-SS-, F127-*g*-Gelatin-TRITC, and CpG-FITC/BPEI-SS-/F127-*g*-Gelatin-TRITC at FITC excitation (495 nm)/FITC emission (519 nm), TRITC excitation (547 nm)/TRITC emission (572 nm), or FITC excitation (495 nm)/TRITC emission (572 nm) was recorded using a Synergy H4 microplate reader (BioTek).

### Preparation of CpG/BPEI-SS- Polyplex-Loaded F127-*g*-Gelatin Hydrogels

The formation of thermosensitive hydrogels was confirmed using the vial tilting method, as described previously.^35^ F127-*g*-Gelatin hydrogels (4.5 wt %) formed at 37 °C were ultrarapidly frozen under liquid nitrogen, after which the hydrogel microstructure was visualized with scanning electron microscopy (SEM) using a Hitachi SU-8230 at accelerating voltage 1 kV and 10 μ A emission current. To prepare CpG/BPEI-SS- polyplex-loaded F127-*g-*Gelatin hydrogel (CpG-HG), CpG and BPEI-SS- were mixed at a weight ratio = 0.25 in saline, followed by pipetting and incubating 20 min at room temperature. The resultant CpG/BPEI-SS- polyplex was further mixed with F127-*g*-Gelatin resulting in a solution with a 4.5 wt % concentration of F127-*g*-Gelatin and 6 μg CpG per 30 μL. The resultant solution formed thermosensitive hydrogels at 37 °C. Supernatants released from CpG-HG hydrogels were analyzed by TEM and DLS, as described above. For imaging flow cytometry experiments, FITC labeled F127-*g*-Gelatin was prepared by reacting 8 mg of F127-*g*-Gelatin in 1 mL of PBS with 160 μL of 1 mg mL^–1^ FITC in DMSO at room temperature for 2 h and then purifying samples with Amicon Ultra centrifugal filters (Milipore, MWCO 30 kDa) at 4000 g and 4 °C for 20 min. CpG/BPEI-SS- polyplex loading was then performed as described above.

### *In Vitro* Residence Stability and Release of CpG from Hydrogels

300 μL of CpG-FITC-containing CpG-HG hydrogels (solvent = PBS, 4.5 wt % F127-*g*-Gelatin, total CpG-FITC = 15 μg, CpG-FITC/BPEI-SS- (w/w) = 0.25) were prepared in 1.5 mL e-tube in a 37 °C incubator. An additional 300 μL of PBS (37 °C) with or without 2.5 U mL^–1^ MMP-9 was added to the e-tube as a supernatant. After supernatants were sampled with a pipet at predetermined time intervals, their CpG-FITC fluorescence was quantified using a Synergy H4 microplate reader, and the remaining hydrogel masses were recorded. 300 μL of fresh PBS with or without 2.5 U mL^–1^ MMP-9 was then added at the end of every time point. The cumulative release of CpG was then calculated based on the supernatant volume.

### Cell Lines

Murine B16F10 cell lines were maintained in Dulbecco’s modified Eagle’s medium, low glucose, GlutaMAX Supplement, and pyruvate (DMEM, ThermoFisher Scientific) supplemented with 10% heat-inactivated fetal bovine serum (HI-FBS, ThermoFisher Scientific) and 1% penicillin/streptomycin/amphotericin B (PSA, ThermoFisher Scientific) in incubators set to 37C with 5% CO_2_, and were passaged at ∼80% confluency. Cell lines did not exceed 25 passages.

### Animal Ethics

All animal studies were approved by the Georgia Institute of Technology’s Institutional Animal Care and Use Committee (IACUC) under protocol numbers A100379 and A100305. C57Bl/6 mice purchased from Jackson Laboratories were housed in ventilated cages (max 5 mice/cage) supplying food and water. UBC-PA-GFP mice purchased from Jackson Laboratories were bred in house and housed as described above. Housing conditions were maintained at a 12 h light/12 h dark cycle, 22 °C, and 41% humidity. Animals were randomized into experimental groups on the day of the first treatment. Humane end points for mice include severely hunched appearance, more than 10% body weight loss, or tumor size larger than 1.5 cm in any dimension. During experiments, animals were anesthetized using isoflurane and were euthanized using CO_2_.

### *In Vivo* Cell Migration Studies

For experiments with FTY-720 (Cayman Chemical), 10^5^ B16F10 cells were suspended in 30 μL of saline and then intradermally (i.d.) implanted in the lateral dorsal skin of C57Bl/6 mice (female, 6–8 weeks old) on day 0. Mice were treated intraperitoneally (i.p.) with 25 μg of FTY-720 dissolved in 20 μL sterile DMSO (VWR) diluted to 100 μL in sterile saline and sonicated until homogeneous, or alternatively 100 μL of 20% DMSO vehicle, on day 7 and 9. Blood was drawn via facial vein laceration on day 8. For experiments with LN photoactivation, 10^5^ B16F10 cells were suspended in 30 μL of saline and then intradermally implanted in the lateral dorsal skin of PA-GFP mice on day 0. On day 4, mice were anesthetized and a small incision was made in the skin over the tumor-draining brachial lymph node using sterile scissors. The tumor was covered with a piece of aluminum foil to prevent inadvertent photoactivation of immune cells in the tumor. The brachial lymph node was then exposed to 405 nm light from an LED (ThorLabs) for 5 min while being kept moistened with sterile prewarmed saline. Immediately afterward, the skin was closed with sterile wound clips and sustained release buprenorphine was administered as an analgesic. ICB treated mice received a single i.p. injection of 150 μg aPD-1 and 150 μg aCTLA-4 in 40 μL total volume after analgesic. On day 5, mice were sacrificed and tumors collected for flow cytometry analysis.

### *In Vivo* Biodistribution of CpG

10^5^ B16F10 cells were suspended in 30 μL of saline and then intradermally implanted in the lateral dorsal skin of C57Bl/6 mice (female, 6–8 weeks old) on day 0. Thirty μL of either free CpG-Cy5.5, CpG-Cy5.5/BPEI-SS- (CpG-BPEI), or CpG-Cy5.5/BPEI-SS-/F127-*g*-Gelatin (CpG-HG) (final concentration of F127-*g*-Gelatin hydrogel = 4.5 wt %. CpG dose equivalent to 6 μg per mouse) was administered i.d. to the forelimb ipsilateral (i.l.) to the tumor on day 4. TdLNs, NdLNs, spleen, and tumor were harvested after mice were sacrificed on days 5, 7, and 11, after which fluorescence images of the tissues were obtained and quantified using an IVIS Spectrum (PerkinElmer). Tissues were then processed for flow cytometry analysis. For imaging flow cytometry experiments, 30 μL of either free CpG, CpG-BPEI, or CpG-HG (CpG labeled with Cy5.5, HG labeled with FITC, CpG dose equivalent to 6 μg per mouse) was administered i.d. in the i.l. forelimb on day 4. TdLNs were harvested after mice were sacrificed on day 5 and were processed for imaging flow cytometry.

### *In Vivo* Therapeutic Studies

10^5^ B16F10 cells were suspended in 30 μL saline and then intradermally implanted in the lateral dorsal skin of C57Bl/6 mice (female, 6–8 weeks old) on day 0. For the ICB therapy experiment, 30 μL of either saline, free CpG, blank F127-*g*-Gelatin hydrogel (blank HG), or CpG-HG (final concentration of F127-*g*-Gelatin hydrogel = 4.5 wt %, CpG was dose equivalent to 6 μg per mouse) was administered i.d. to the forelimb i.l. to the tumor on day 4. aCTLA-4 (150 μg per mouse) and aPD-1 (150 μg per mouse) mAbs or isotype mAbs were administered i.p. on day 5, 7, 9, 11, 13, 15, and 18. For the ICB dose reduction experiment, 30 μL of either saline, free CpG, or CpG-HG (final concentration of F127-*g*-Gelatin hydrogel = 4.5 wt %, CpG was dose equivalent to 6 μg per mouse) was administered i.d. to the forelimb i.l. to the tumor on day 4. aCTLA-4 (150 μg per mouse) and aPD-1 (150 μg per mouse) mAbs were administered i.p. either on day 5, 7, and 9 or on day 5 only. Tumor sizes were recorded by measuring the dimensions of the tumors with calipers and then calculating an ellipsoidal volume (*V* = (π/6) × *abc*, where *a* is height, *b* is width, and *c* is length, respectively). Average tumor growth curves representing mean ± SEM from multiple animals are presented while more than 50% of mice are alive in a group. Animal survival is presented by Kaplan–Meier curves. Animal body weight is presented as body weight relative to body weight on day 4 after tumor implant.

### *In Vivo* Peripheral Blood Immune Profiling

10^5^ B16F10 cells were suspended in 30 μL saline and then intradermally implanted in the lateral dorsal skin of C57Bl/6 mice (female, 6–8 weeks old) on day 0. For immune profiling experiments without ICB, blood was drawn via facial vein laceration on days 6, 9, and 11 after tumor implant from mice treated with saline, free CpG, blank F127-*g*-Gelatin hydrogel (blank HG), or CpG-HG (final concentration of F127-*g*-Gelatin hydrogel was 4.5 wt %. CpG was dose equivalent to 6 μg per mouse) on day 4 after tumor implant. For immune profiling experiments with ICB, blood was drawn via facial vein laceration on days 6, 7, 9, 11, and 13 after tumor implant from mice treated with saline, free CpG, blank F127-*g*-Gelatin hydrogel (blank HG), or CpG-HG (final concentration of F127-*g*-Gelatin hydrogel was 4.5 wt %. CpG was dose equivalent to 6 μg per mouse) on day 4 after tumor implant and with aCTLA-4 (150 μg per mouse) and aPD-1 (150 μg per mouse) mAbs or isotype mAbs on day 5.

### *In Vivo* ALT/AST Analysis

10^5^ B16F10 cells were suspended in 30 μL saline and then intradermally implanted in the lateral dorsal skin of C57Bl/6 mice (female, 6–8 weeks old) on day 0. Mice were treated with saline, free CpG, blank F127-*g*-Gelatin hydrogel (blank HG), or CpG-HG 4 days after tumor implant followed aCTLA-4 (150 μg per mouse) and aPD-1 (150 μg per mouse) mAbs or isotype mAbs on day 5. Blood was drawn via facial vein laceration into tubes containing EDTA on day 6 and plasma was harvested from blood after 2× centrifugation at 2100*g* for 10 min. Alanine aminotransferase (ALT) activity colorimetry/fluorometry (Biovision) and aspartate aminotransferase (AST) activity colorimetric assay kits (Biovision) were used to determine ALT and AST activity and were performed per the manufacturer’s instructions.

### Flow Cytometry Analysis

Following dissection, lymph nodes and tumors were incubated in collagenase D (1 mg mL^–1^) at 37 °C for 75 min and 4 h, respectively. Leukocytes were harvested by passing the tissues, including TdLNs, NdLNs, tumors, and spleen, through 70 μm strainers and washing with ice-cold PBS 1× +/+. Leukocytes obtained from spleens and blood samples were incubated with Red Blood Cell Lysing Buffer Hybri-Max for 7 and 12 min, respectively, at room temperature, followed by quenching and washing with ice-cold PBS. Single cell suspensions in PBS were plated in 96 well U-bottom plates for staining. Leukocytes were blocked with Fc block (2.4G2) on ice for 5 min and then stained with Zombie Aqua or Zombie UV fixable viability dye at room temperature for 30 min followed by antibody mixtures for surface staining on ice for 30 min. For experiments involving only surface staining, cells were then fixed using 2% paraformaldehyde (ThermoFisher Scientific) on ice for 15 min. For experiments involving nuclear/intracellular staining, cells were fixed with Fixation/Permeabilization working solution (eBioscience, TM Foxp3/Transcription Factor Staining Buffer Set, Invitrogen TM) on ice for 60 min before being incubated with antibody mixtures against nuclear/intracellular markers on ice for 90 min. At the end of each step, cells were washed with PBS, FACS buffer, or Foxp3 Fixation/Permeabilization washing solution. Finally, cells were resuspended in FACS buffer and analyzed using a Cytek Aurora flow cytometer and FlowJo. For imaging flow cytometry, cells were stained after Fc block with antibody mixtures for surface staining on ice for 30 min. Cells were then incubated with Hoechst 33342 (ThermoFisher Scientific) for nuclear staining for 10 min. Cells were then fixed using 2% paraformaldehyde (ThermoFisher Scientific) at room temperature for 20 min. At the end of each step, cells were washed with PBS or FACS buffer. Finally, cells were resuspended in FACS buffer and analyzed using an Amnis Imagestream Mk II imaging flow cytometer. Data acquired on the Imagestream were analyzed using FlowJo and IDEAS imaging flow cytometry software.

### Statistical Analysis

*In vitro* and *in vivo* data are presented as mean ± standard deviation (SD), or mean ± standard error of the mean (SEM), respectively. GraphPad Prism v10 was used for plotting graphs and for statistically analyzing the data with linear regressions, two-tailed unpaired *t* tests, and one-way or two-way ANOVA with Tukey’s posthoc test for multiple comparisons, as well as with log-rank analysis with Mantel-Cox statistical hypothesis for survival data. IBM SPSS Statistics was used for classification and regression tree (CART) analyses. ****p < 0.0001, ***p < 0.001, **p < 0.01, and *p < 0.05.

## Supplementary Material



## Notes

Schematic indicating anatomical sites of administration used in *in vivo* experiments (Figure S1); synthetic scheme for and ^1^H NMR of BPEI-SS- (Figures S2 and S3); ζ-potential of CpG, BPEI-SS-, F127-*g*-Gelatin, CpG/BPEI-SS, and CpG-HG (Figure S4); fluorescence spectra of CpG-FITC, BPEI-SS, and CpG-FITC/BPEI-SS (Figure S5); synthetic scheme for and ^1^H NMR of F127-*g*-Gelatin (Figures S6 and S7); fluorescence measurements from FRET assays (Figure S8); ζ-potential of polyplex micelles released *in situ* from CpG-HG hydrogels (Figure S9); correlation of CpG-FITC release with F127-*g*-Gelatin hydrogel degradation (Figure S10); flow cytometry gating strategy for *in vivo* CpG biodistribution and immunomodulation (Figure S11); additional IVIS and flow cytometry readouts of *in vivo* CpG biodistribution and immunomodulation (Figures S12–S16); *P* values for significant comparisons from 2-way ANOVA in Figure 6A–C (Figure S17); ALT and AST activity from CpG-HG and ICB treated mice (Figure S18); and antibodies used for flow cytometry (Table T1) (PDF)

## ∇

PPD, Inc., Waltham, Massachusetts 02451, United States

## ○

Takeda Pharmaceuticals, Cambridge, Massachusetts 02139, United States.

## Abbreviations

LN: lymph node
dLN: draining lymph node
TdLN: tumor-draining lymph node
NdLN: nondraining lymph node
ICB: immune checkpoint blockade
PD-1: programmed cell death protein 1
CTLA-4: cytotoxic T lymphocyte associated protein 4
TLR: toll like receptor
APC: antigen presenting cell
DC: dendritic cell
BPEI-SS-: disulfide-cross-linked polyethylenimine
F127-*g*-Gelatin: Pluronic F127-grafted gelatin polymer
CpG-HG: CpG/BPEI-SS-F127-*g*-Gelatin
i.p.: intraperitoneal
FTY: FTY720
PA-GFP+: photoactivated
MΦ: macrophage
MFI: median fluorescence intensity
