# Supplementary material for: Thermosensitive Hydrogel Sustaining the Release of Lymph-Draining Oligonucleotide Adjuvant Polyplex Micelles Improves Systemic Cancer Immunotherapy
Source: ACS Nano. 2025 Jun 3;19(23):21775–91. doi: 10.1021/acsnano.5c05517 (PMC12177939; doi:10.1021/acsnano.5c05517)
Supplement: Supplementary file 1 [file nn5c05517_si_001.pdf]

Thermosensitive Hydrogel Sustaining the Release of Lymph-Draining Oligonucleotide Adjuvant Polyplex Micelles Improves Systemic Cancer Immunotherapy

Samuel N. Lucas[1], Paul A. Archer[2,3†], Tae Hee Yoon[2,4], Margaret P. Manspeaker[2,3††], Maya Levitan[1], Jihoon Kim[5], Susan N. Thomas[1,2,4,6]**

[1]Wallace H. Coulter Department of Biomedical Engineering, Georgia Institute of Technology and Emory University, Atlanta, GA, 30332, United States of America

[2]Parker H. Petit Institute for Bioengineering and Bioscience, Georgia Institute of Technology, Atlanta, GA 30332, United States of America

[3]School of Chemical and Biomolecular Engineering, Georgia Institute of Technology, Atlanta, GA 30332, United States of America

[4]George W. Woodruff School of Mechanical Engineering, Georgia Institute of Technology, Atlanta, GA, 30332, United States of America

[5]School of Integrative Engineering, Chung-Ang University, Seoul 06974, South Korea

[6]Winship Cancer Institute, Emory University, Atlanta, GA, 30322, United States of America

Supporting Fig S1

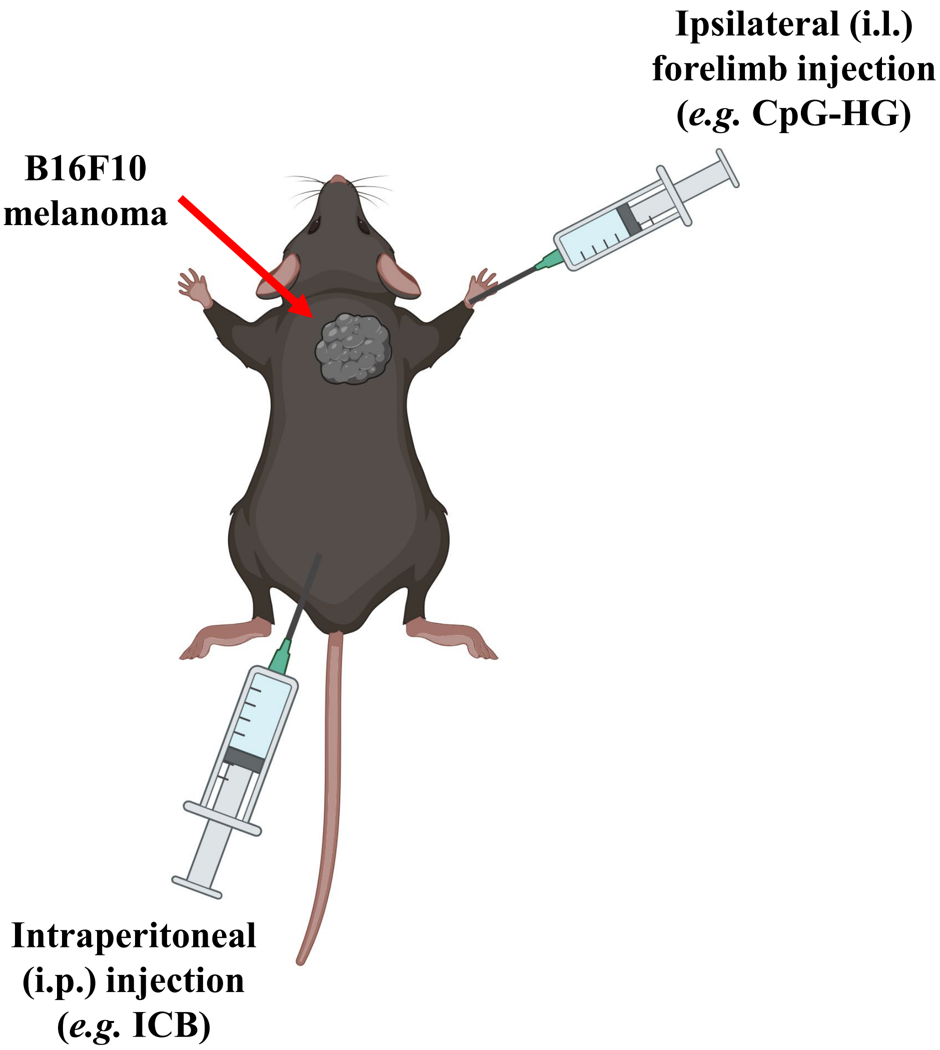

Figure S1. Anatomical sites of administration. B16F10 melanoma-bearing mice received injections of saline, free CpG, CpG/BPEI-SS-, or CpG-HG in the ipsilateral (i.l.; accessed by the TdLN) forelimb skin and injections of FTY720, ICB antibodies, or isotype antibodies intraperitoneally (i.p.; systemic administration).

Supporting Fig S2

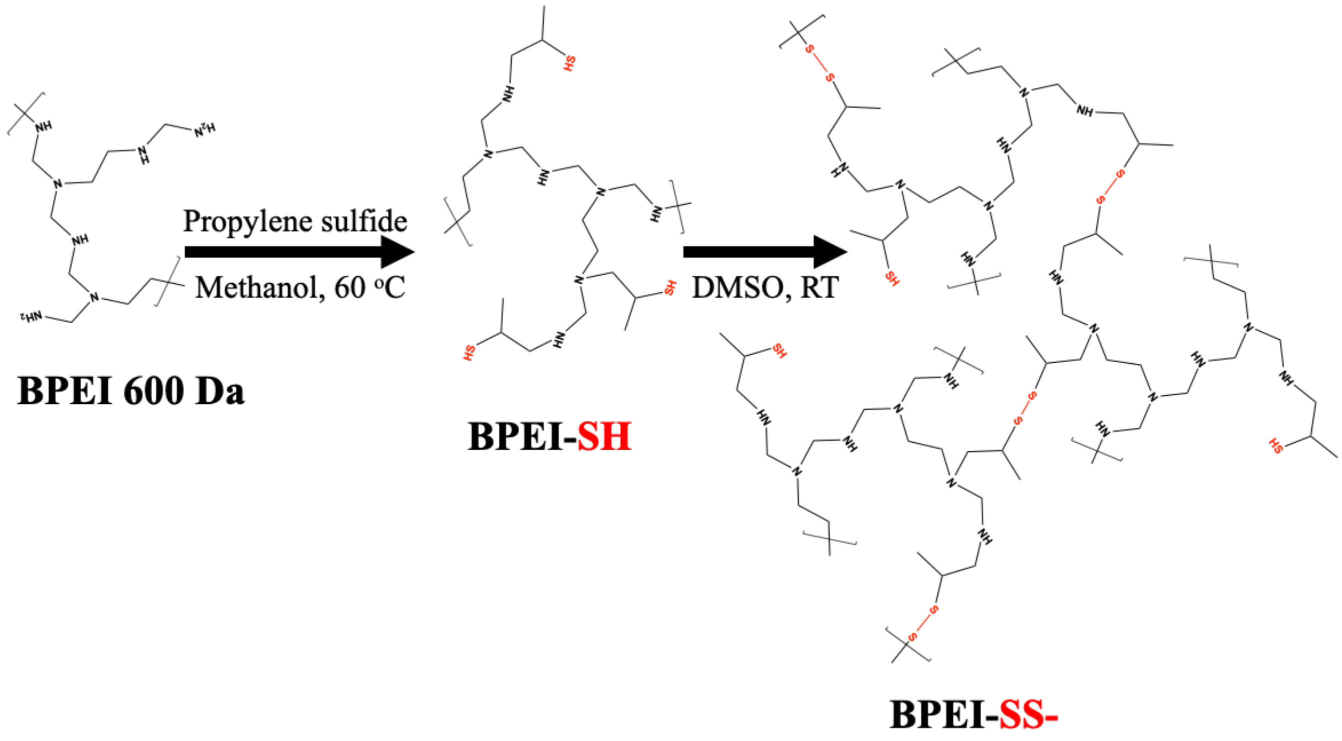

Figure S2. Synthetic scheme for BPEI-SS-. Primary and secondary amines of BPEI were converted to thiol groups using propylene sulfide before free thiols were crosslinked in oxidative conditions in DMSO.

Supporting Fig S3

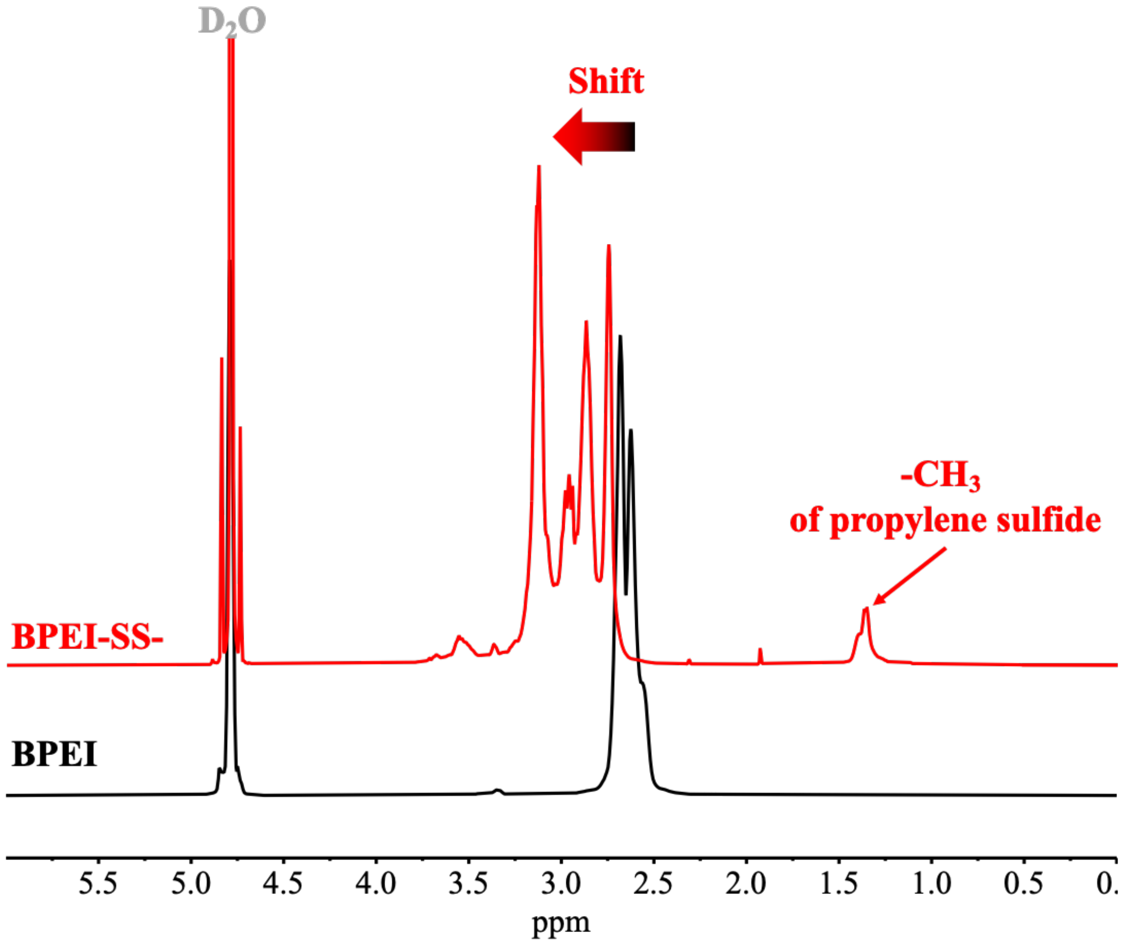

Figure S3. ^{1}H NMR of BPEI-SS- in D_2O using Bruker Avance 400 MHz FT-NMR. Methyl groups from propylene sulfide (δ 1.2 – 1.5 ppm) and peak-shifts and-broadening (δ 2.6–3.3 ppm) of ethyl protons adjacent to the primary, secondary, and tertiary amines of BPEI in BPEI-SS.

<u>Supporting Fig S4</u>

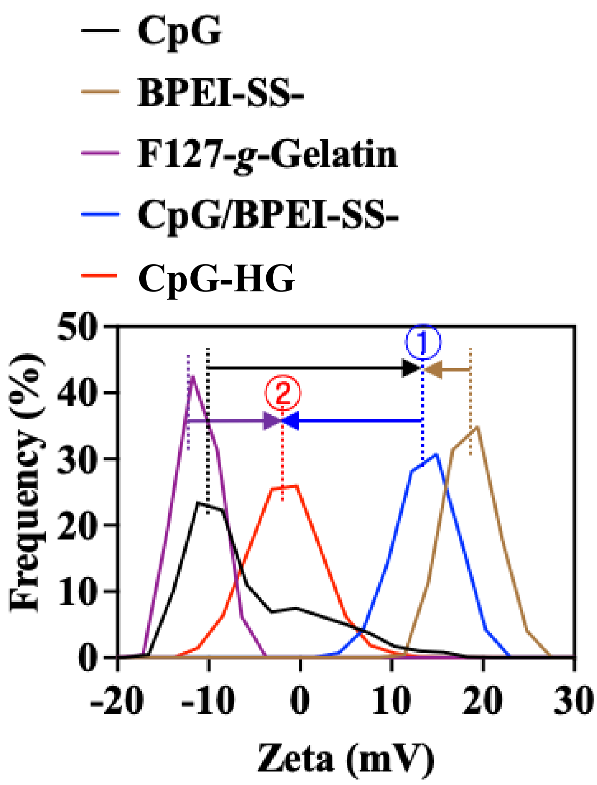

Figure S4. Zeta potential distribution of CpG, BPEI-SS-, F127-*g*-Gelatin micelles, CpG/BPEI-SS- polyplex, and CpG-HG polyplex micelles. ① Zeta potential of CpG/BPEI-SS- polyplexes made by mixing CpG and BPEI-SS-. ② Zeta potential of CpG-HG polyplex micelles made by mixing CpG/BPEI-SS- polyplex and F127-*g*-Gelatin. N = 6.

Supporting Fig S5

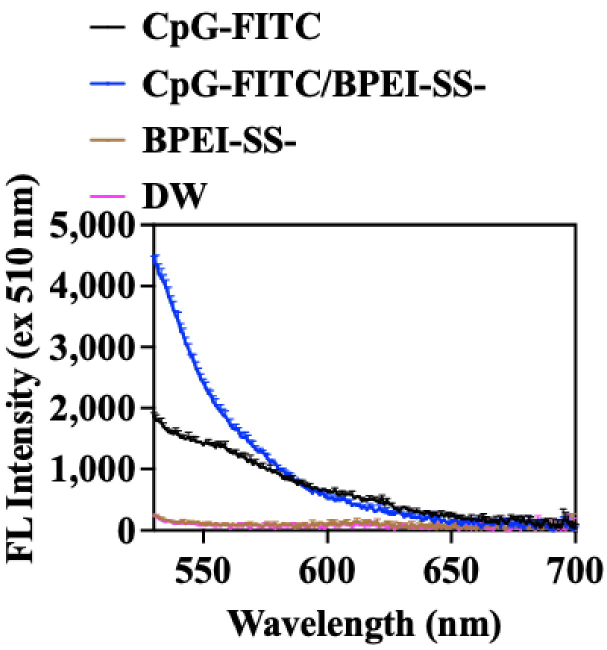

Figure S5. Fluorescence spectra of CpG-FITC, BPEI-SS-, and CpG-FITC/BPEI-SS- polyplexes at an excitation wavelength (510 nm) where CpG-FITC/BPEI-SS- polyplexes have maximum absorbance. N = 3. Data are presented as mean ± SD.

Supporting Fig S6

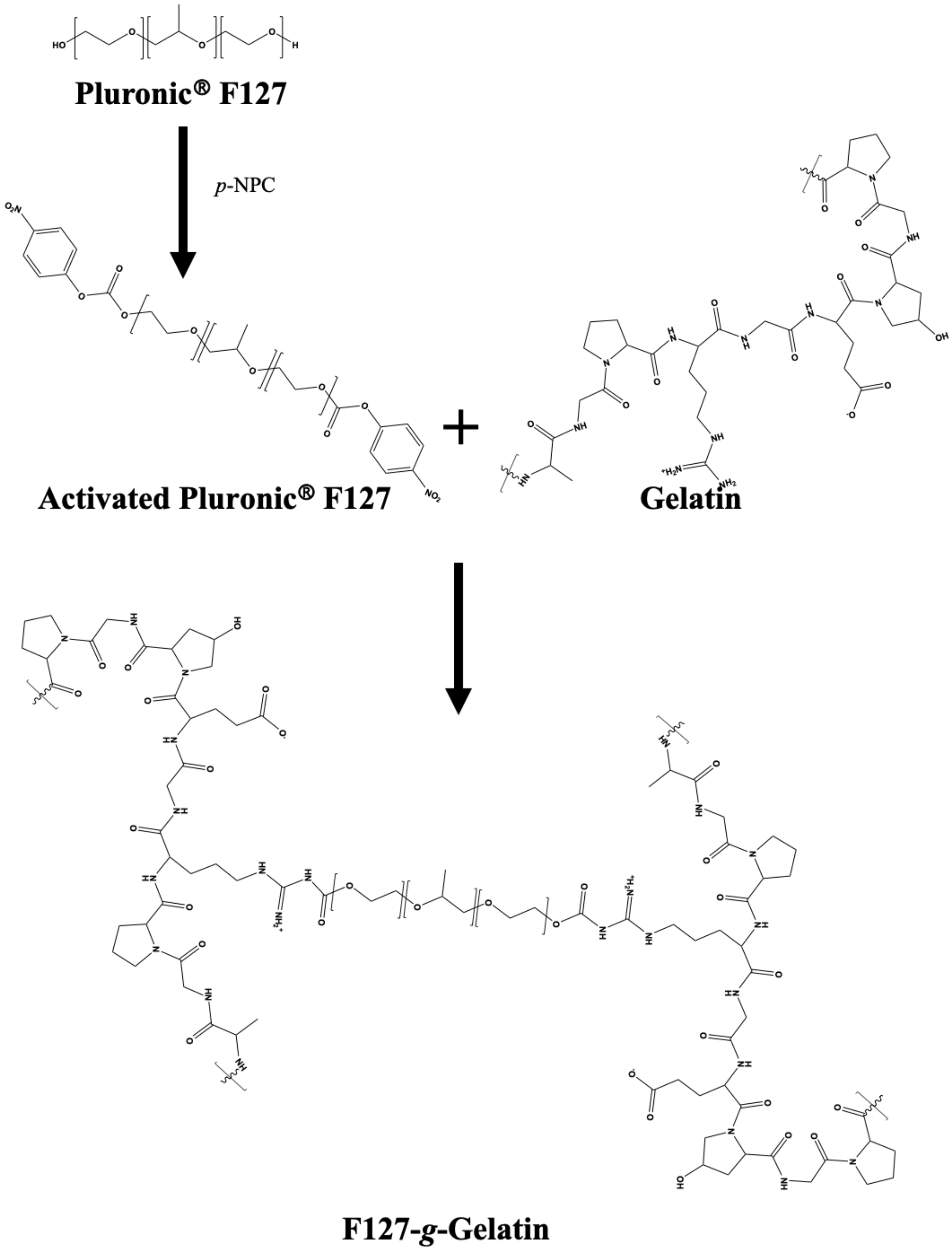

Figure S6. Synthetic scheme for F127-*g*-Gelatin. Hydroxyl groups of Pluronic® F127 were activated with 4-nitrophenyl chloroformate (*p*-NPC) followed by reaction with the amine groups of gelatin to yield F127-*g*-Gelatin polymers.

Supporting Fig S7

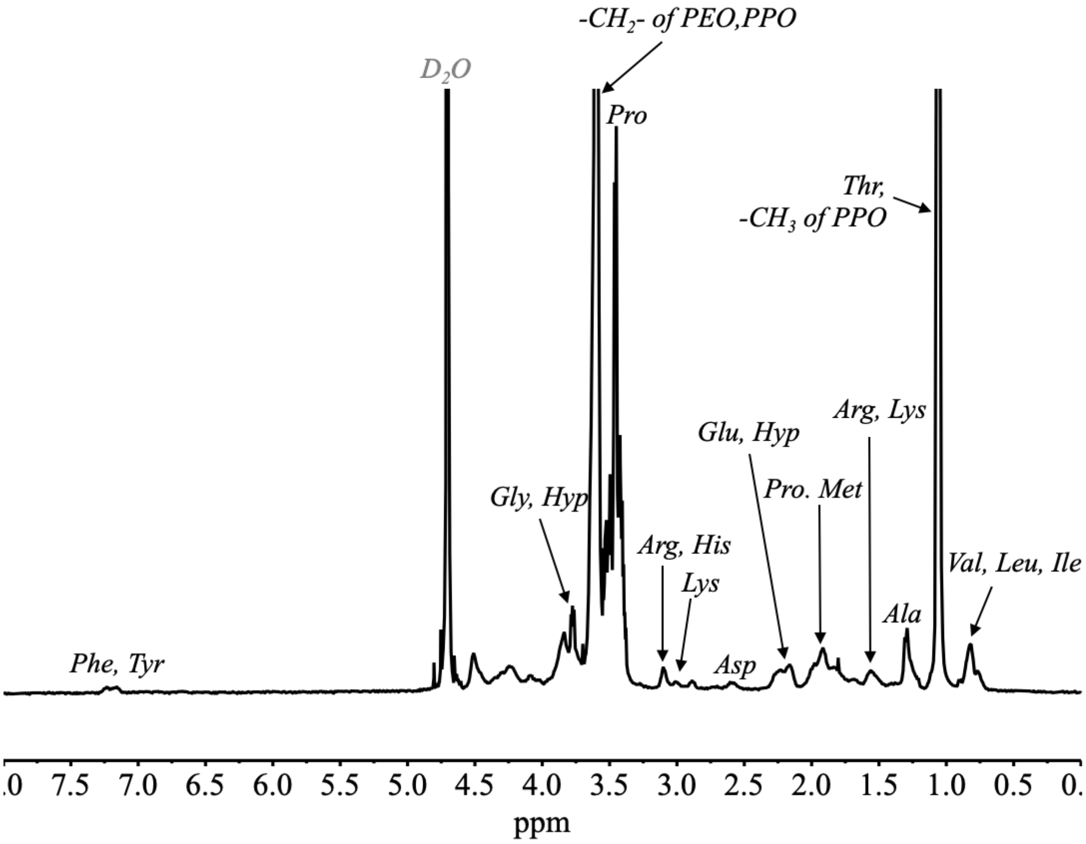

Figure S7. ^{1}H NMR of F127-*g*-Gelatin in D_2O using Bruker Avance 400 MHz FT-NMR. The characteristic peaks of gelatin (Phe, Tyr, Gly, Hyp, Pro, Arg, His, Lys, Asp, Met, Thr, Val, Leu, and Ile) and Pluronic® F127 (δ 1.0–1.2 ppm for methyl groups, δ 3.6–3.7 ppm for -CH2- of polyethylene oxide and polypropylene oxide) are annotated.

Supporting Fig S8

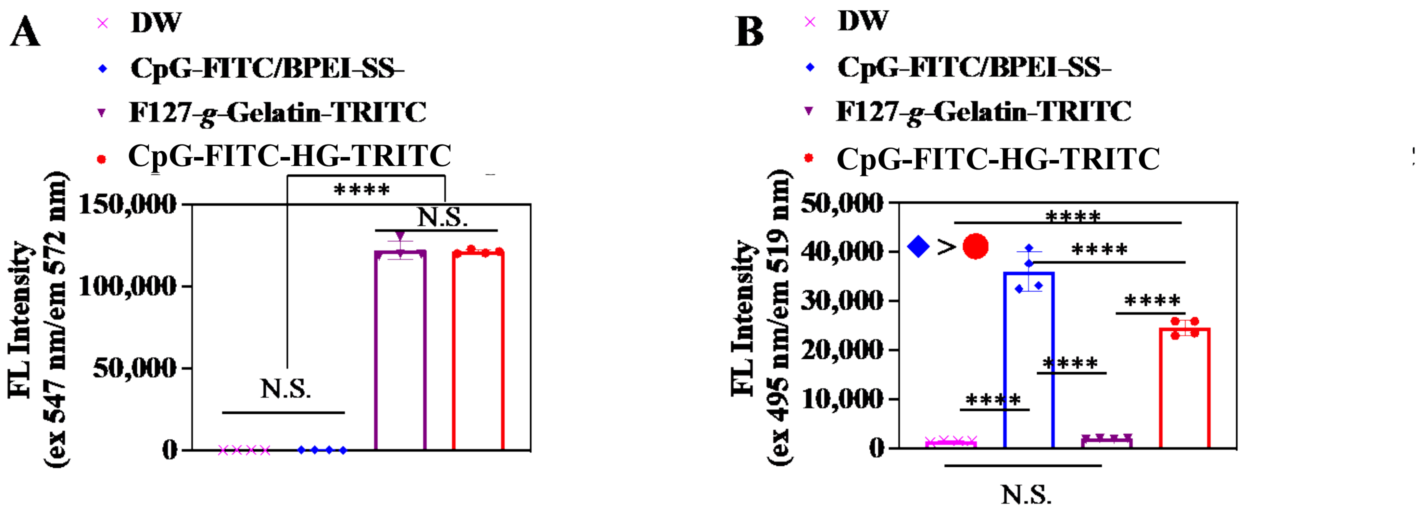

Figure S8. Fluorescence intensity of CpG-FITC/BPEI-SS- polyplexes, F127-*g*-Gelatin-TRITC, and CpG-FITC-HG-TRITC at A) TRITC excitation (547 nm) and emission (572 nm), and B) FITC excitation (495 nm) and emission (519 nm). N = 4. Data are presented as mean ± SD. ****p<0.0001, ***p<0.001, **p<0.01, *p<0.05 One-way ANOVA using Tukey's test.

Supporting Fig S9

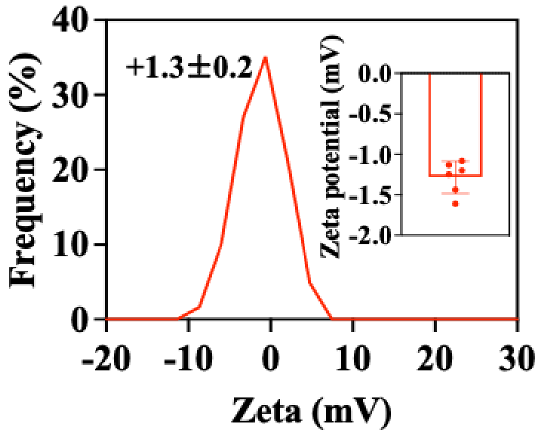

Figure S9. Zeta potential distribution of CpG-HG polyplex micelles released *in situ* from F127-*g*-Gelatin hydrogels containing CpG/BPEI-SS-. N = 6. Data are presented as mean ± SD.

Supporting Fig S10

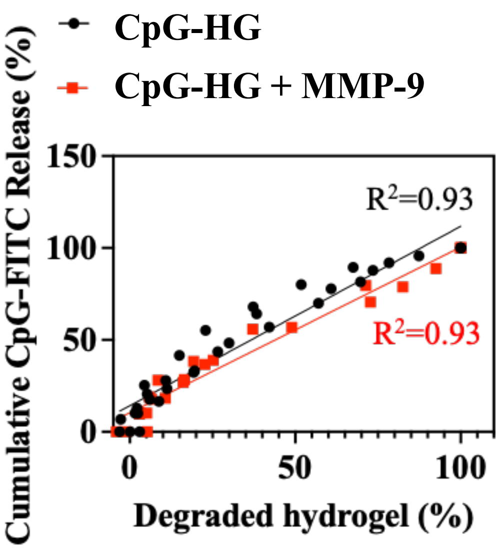

Figure S10. Correlation of CpG-FITC release with F127-*g*-Gelatin hydrogel degradation. N = 3.

Supporting Fig S11

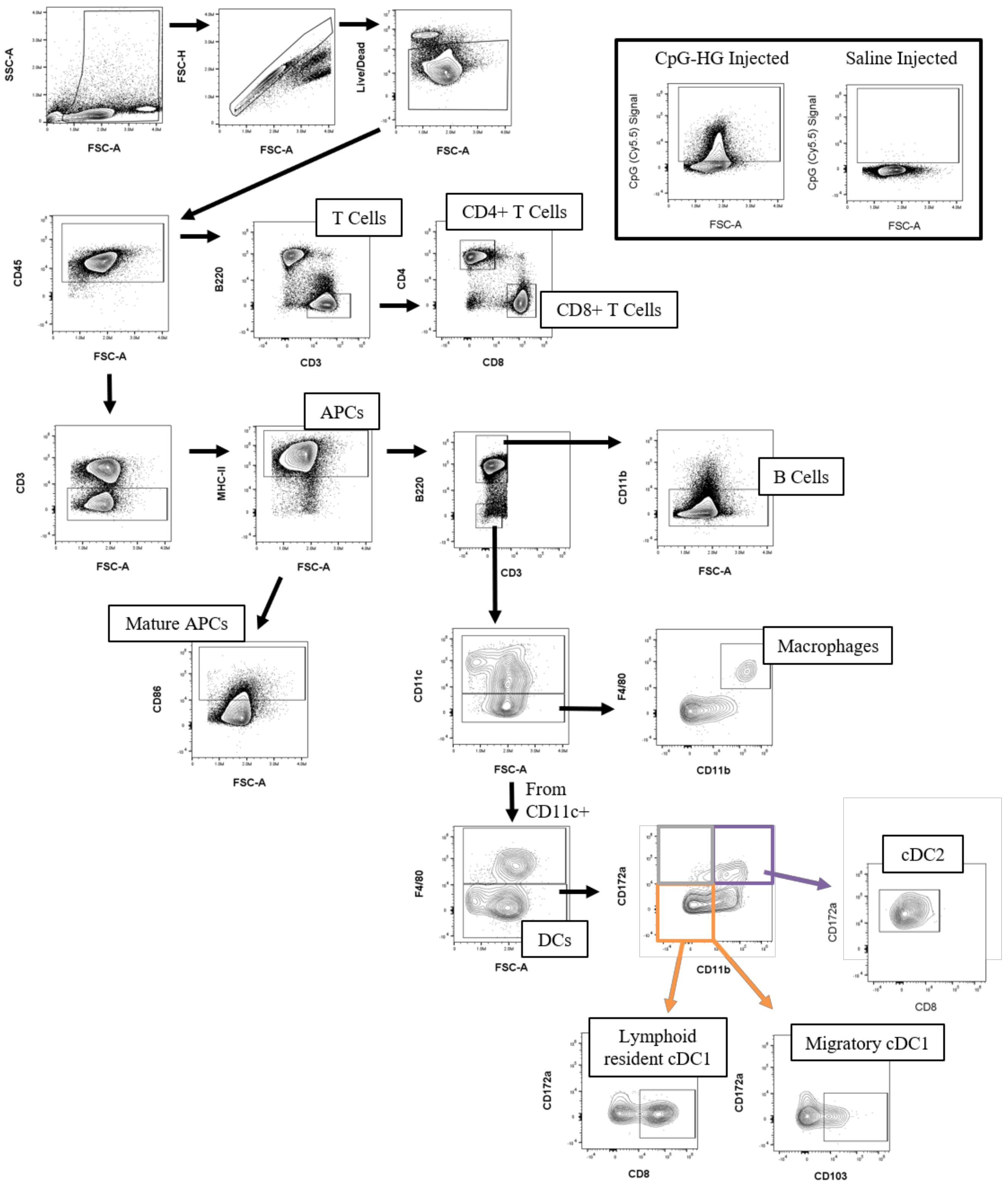

Figure S11. Lymphocyte gating strategy for CpG biodistribution and immunomodulation within lymphoid tissues.

Supporting Fig S12

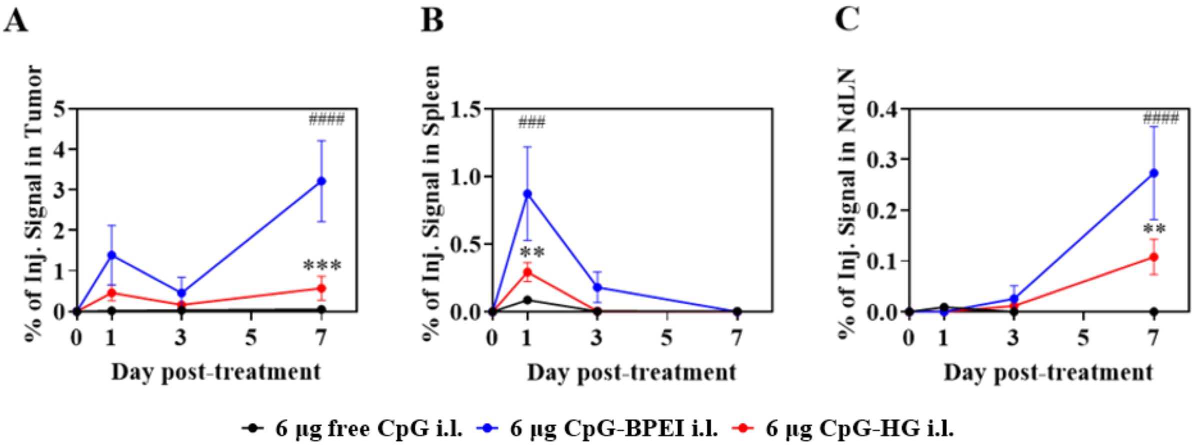

Figure S12. *In vivo* CpG biodistribution over time within tumor, spleen, and NdLN. Mice received 6 µg of CpG in either free, CpG-BPEI, or CpG-HG form in the i.l. forelimb skin and tissues were collected for IVIS analysis 1 day, 3 days, and 7 days after CpG treatment. Quantification (as a % of initial signal at the injection site) of Cy5.5-labeled CpG using IVIS at the A) tumor, B) spleen, and C) ndLN over time. N = 5. Data are presented as mean ± SEM. $^{\#\#\#\#}p<0.0001$, $^{\#\#\#}p<0.001$, $^{\#\#}p<0.01$, $^{\#}p<0.05$ compared to free CpG. ****$p<0.0001$, ***$p<0.001$, **$p<0.01$, *$p<0.05$ compared to CpG-BPEI. Two-way ANOVA using Tukey's test.

Supporting Fig S13

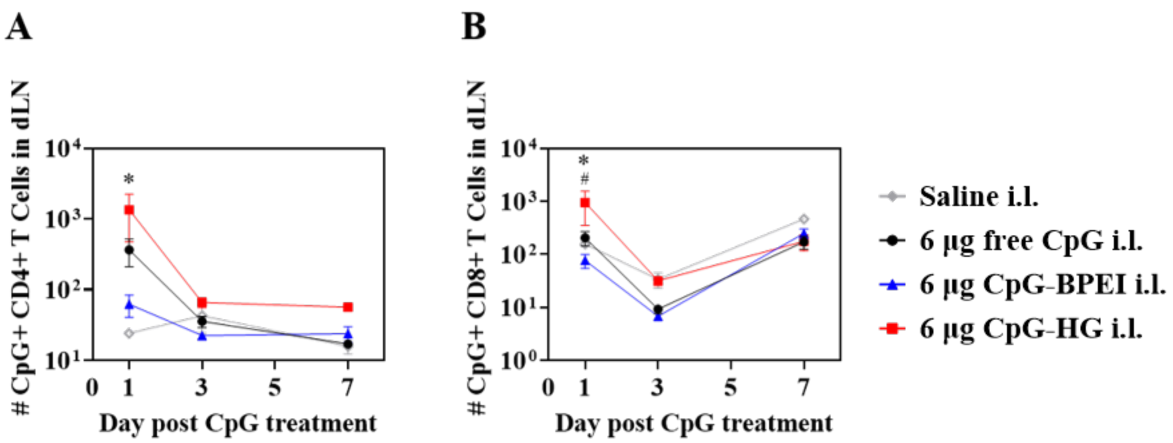

Figure S13. *In vivo* CpG delivery to T cells in the dLN. Mice received 6 µg of CpG in either free, CpG-BPEI, or CpG-HG form in the i.l. forelimb skin and tissues were collected for flow cytometry analysis 1 day, 3 days, and 7 days after CpG treatment. # of CpG+ CD4+ (A) and CD8+ (B) T cells in dLNs over time. N = 5. Data are presented as mean ± SEM. $^{\#\#\#\#}p<0.0001$, $^{\#\#\#}p<0.001$, $^{\#\#}p<0.01$, $^{\#}p<0.05$ compared to free CpG. $^{****}p<0.0001$, $^{***}p<0.001$, $^{**}p<0.01$, $^{*}p<0.05$ compared to CpG-BPEI. Two-way ANOVA using Tukey's test.

Supporting Fig S14

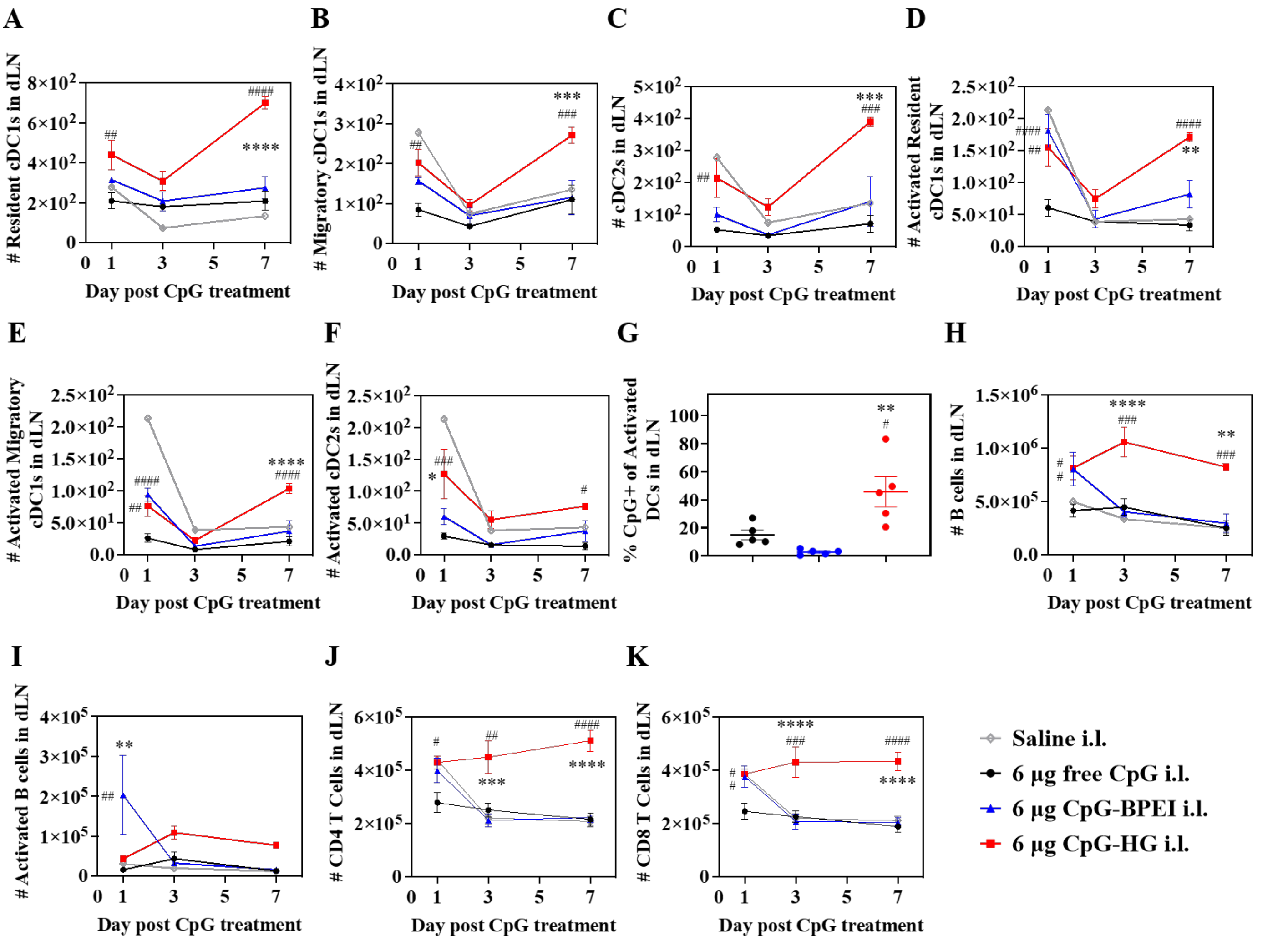

Figure S14. Activation and expansion of lymphocytes in dLNs. Mice received 6 µg of CpG in either free, CpG-BPEI, or CpG-HG form in the i.l. forelimb skin and tissues were collected for flow cytometry analysis 1 day, 3 days, and 7 days after CpG treatment. A-F): # of A) resident cDC1s, B) migratory cDC1s, C) cDC2s, D) activated resident cDC1s, E) activated migratory cDC1s, and F) activated cDC2s in dLNs. G) % of activated DCs in dLNs that are CpG+ 1 day after CpG treatment. H-K): # of H) B cells, I) activated B cells, J) CD4+ T cells, and K) CD8+ T cells in dLNs. N = 5. Data are presented as mean ± SEM. $^{####}p<0.0001$, $^{###}p<0.001$, $^{##}p<0.01$, $^{#}p<0.05$ compared to free CpG. $^{****}p<0.0001$, $^{***}p<0.001$, $^{**}p<0.01$, $^{*}p<0.05$ compared to CpG-BPEI. Two-way ANOVA using Tukey's test.

Supporting Fig S15

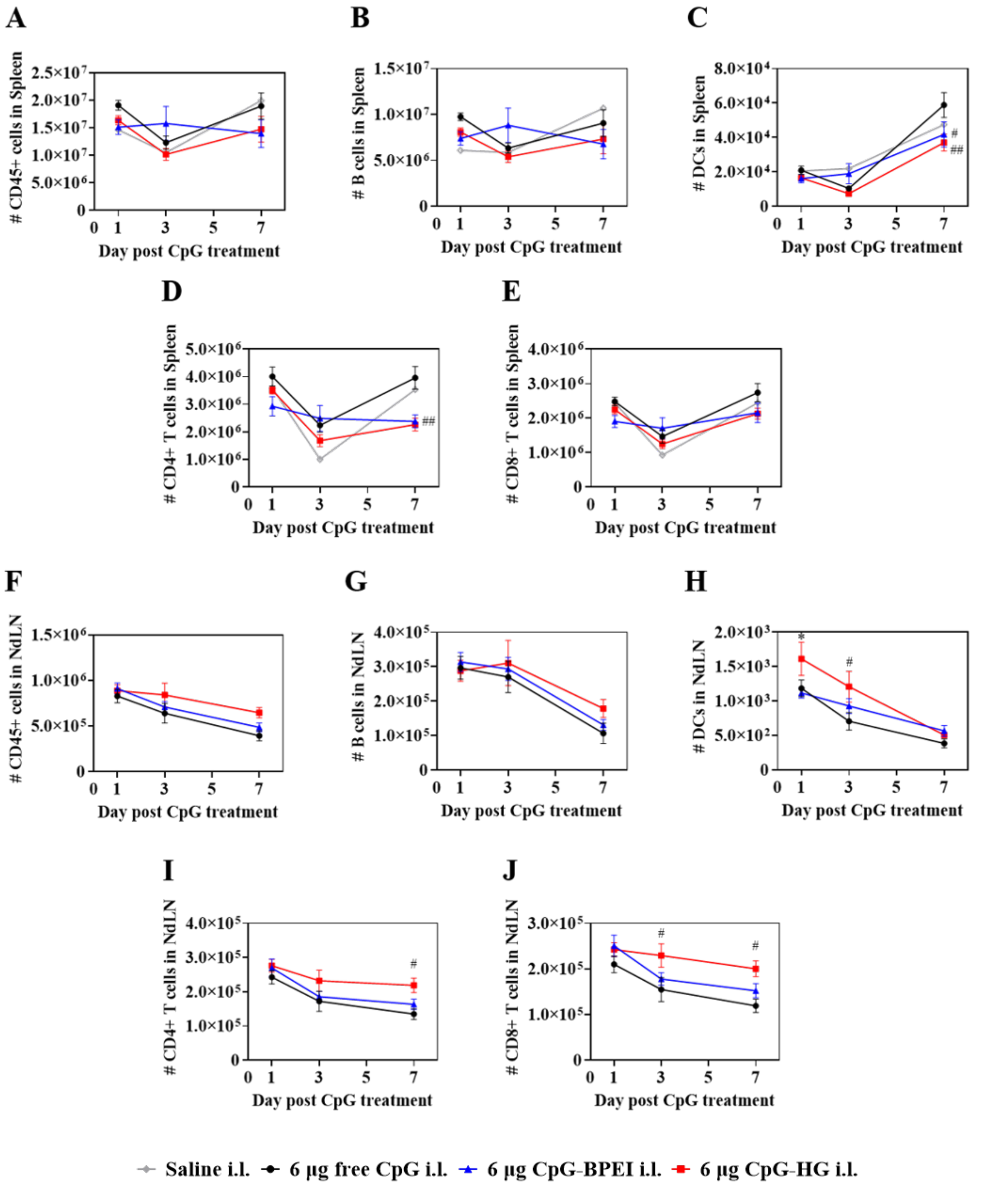

Figure S15. Immune profiling of lymphocytes in spleens and NdLNs. Mice received 6 µg of CpG in either free, CpG-BPEI, or CpG-HG form in the i.l. forelimb skin and tissues were collected for flow cytometry analysis 1 day, 3 days, and 7 days after CpG treatment. A-E) # of A) CD45+ cells, B) B cells, C) DCs, D) CD4+ T cells, and E) CD8+ T cells in spleens. F-J) # of F) CD45+ cells, G) B cells, H) DCs, I) CD4+ T cells, and J) CD8+ T cells in NdLNs. N = 5. Data are presented as mean ± SEM. $^{####}p<0.0001$, $^{###}p<0.001$, $^{##}p<0.01$, $^{#}p<0.05$ compared to free CpG. $^{****}p<0.0001$, $^{***}p<0.001$, $^{**}p<0.01$, $^{*}p<0.05$ compared to CpG-BPEI. Two-way ANOVA using Tukey's test.

Supporting Fig S16

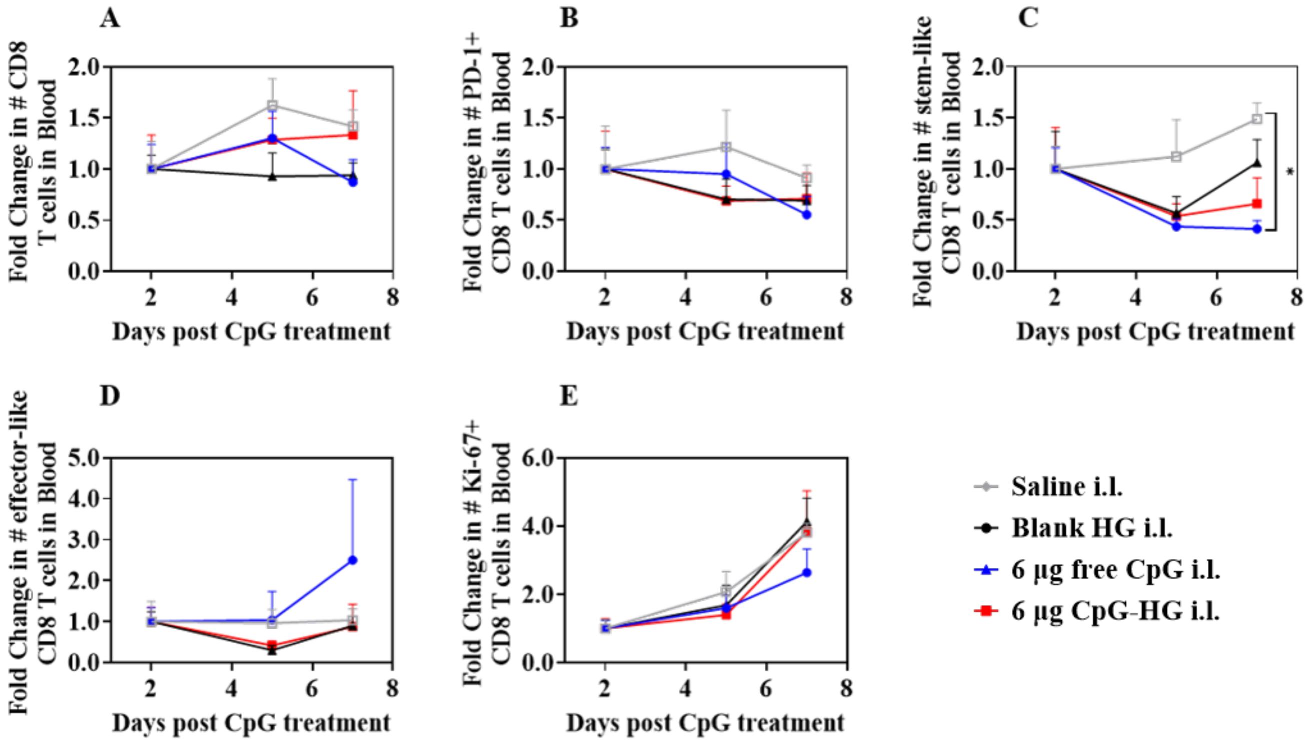

Figure S16. Circulating T cell response to CpG monotherapy. B16F10-bearing mice were treated with either saline, blank HG, 6 µg of free CpG, or 6 µg of CpG-HG in the i.l. forelimb skin 4 days after tumor inoculation. Fold change relative to 2 days after CpG treatment in # of A) CD8+ T cells, B) PD-1+ CD8+ T cells, C) stem-like (PD-1+TCF1+Tim3-) CD8+ T cells, D) effector-like (PD-1+TCF1-Tim3+) CD8+ T cells and E) Ki-67+ T-cells in 100 µL of peripheral blood. N = 5. Data are presented as mean ± SEM. ****p<0.0001, ***p<0.001, **p<0.01, *p<0.05. Two-way ANOVA using Tukey's test.

Supporting Fig S17

A

Day post CpG	Groups	P-value
5	CpG-HG + ICB vs. Saline + Isotype	P = 0.0054
5	CpG-HG + ICB vs. CpG-HG + Isotype	P = 0.0013
7	CpG-HG + ICB vs. all other groups	P < 0.0001
9	CpG-HG + ICB vs. Saline + Isotype	P = 0.0147
9	CpG-HG + ICB vs. Saline + ICB	P = 0.0126
9	CpG-HG + ICB vs. CpG-HG + Isotype	P = 0.0043

B

Day post CpG	Groups	P-value
5	CpG-HG + ICB vs. Saline + Isotype, CpG-HG + Isotype, Blank HG + ICB	P < 0.0001
5	CpG-HG + ICB vs. Saline + ICB	P = 0.0003
5	Free CpG + ICB vs. CpG-HG + Isotype	P = 0.0084
7	CpG-HG + ICB vs. all other groups	P < 0.0001
9	CpG-HG + ICB vs. Saline + ICB, Blank HG + ICB, CpG-HG + Isotype	P < 0.0001
9	CpG-HG + ICB vs. Saline + Isotype	P = 0.0004
9	CpG-HG + ICB vs. Free CpG + ICB	P = 0.0042

C

Day post CpG	Groups	P-value
5	CpG-HG + ICB vs. Saline + Isotype, Saline + ICB, CpG-HG + Isotype	P < 0.0001
5	CpG-HG + ICB vs. Free CpG + ICB	P = 0.002
5	CpG-HG + ICB vs. Blank HG + ICB	P = 0.0026
5	Free CpG + ICB vs. CpG-HG + Isotype	P = 0.0455
5	Blank HG + ICB vs. CpG-HG + Isotype	P = 0.0376
7	CpG-HG + ICB vs. all other groups	P < 0.0001
9	CpG-HG + ICB vs. Saline + ICB, CpG-HG + Isotype	P < 0.0001
9	CpG-HG + ICB vs. Saline + Isotype	P = 0.0001
9	CpG-HG + ICB vs. Free CpG + ICB	P = 0.0044
9	CpG-HG + ICB vs. Blank HG + ICB	P = 0.0123

Figure S17. P values for significant comparisons from two-way ANOVA in Fig 6A-C. A-C) Significant comparisons from A) Fig 6A, B) Fig 6B, and C) Fig 6C by two-way ANOVA using Tukey's test.

Supporting Fig S18

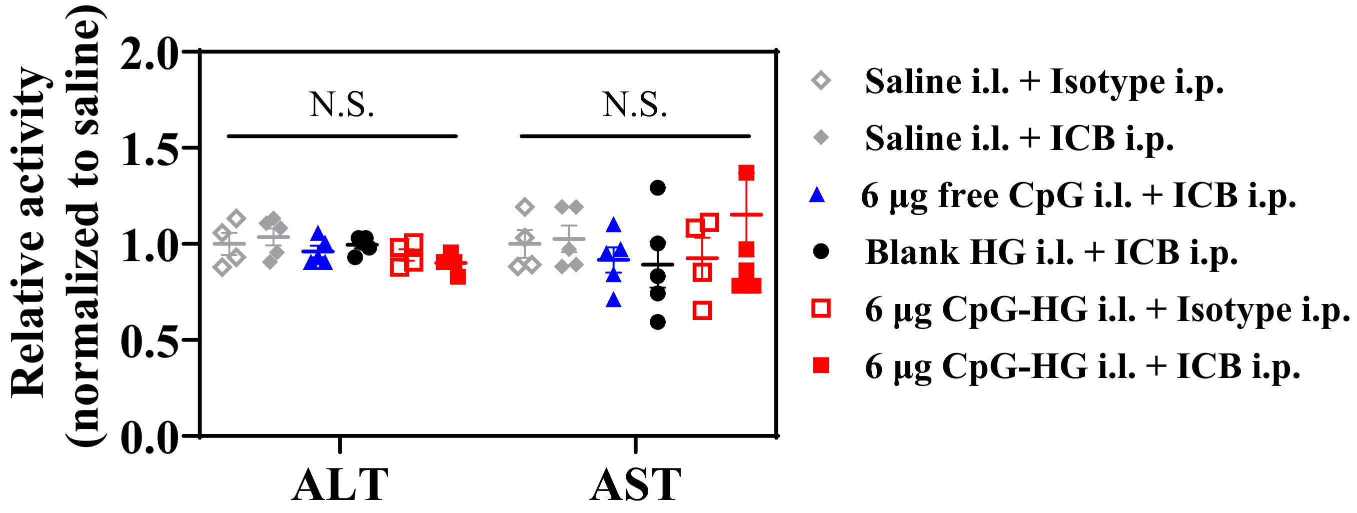

Figure S18. ALT and AST activity in CpG-HG and ICB treated mice. B16F10-bearing mice were treated with saline, blank HG, 6 µg of free CpG, or 6 µg of CpG-HG in the i.l. forelimb skin 4 days after tumor inoculation followed by either 150 µg each i.p. aPD-1 + aCTLA-4 (ICB) or isotype antibodies 5 days after tumor inoculation. Blood plasma was collected 2 days after CpG treatment for ALT/AST analysis. Activity relative to saline of ALT and AST in peripheral blood of B16F10 tumor-bearing mice, 48 hours after i.l. CpG treatment and 24 hours after i.p. ICB treatment. N = 4-6. Data are presented as mean ± SEM. One-way ANOVA using Tukey's test.

Supporting Table T1

Color	Marker	Clone	Company (Cat.#)	Dilution
BV650	B220	RA3-6B2	Biolegend (103241)	1.5:100
PE	CD103	2E7	Biolegend (121406)	1.25:100
PE/Dazzle 594	CD11b	M1/70	Biolegend (101255)	0.3:100
PE/Cy7	CD11c	N418	Biolegend (117318)	0.3:100
APC-Cy7	CD11c	N418	Biolegend (117324)	5:100
FITC	CD172a	P84	Biolegend (144006)	2:100
BV711	CD3	17A2	Biolegend (100241)	1.25:100
BV750	CD4	GK1.5	Biolegend (100467)	0.5:100
APC/Cy7	CD4	RM4-5	Biolegend (100526)	0.1563:100
PerCP	CD45	30-F11	Biolegend (103130)	0.5:100
AF700	CD45	30-F11	Biolegend (103128)	0.625:100
PerCP-Cy5.5	CD45	30-F11	Biolegend (103132)	0.625:100
APC/Cy7	CD8	53-6.7	Biolegend (100714)	0.5:100
FITC	CD8	53-6.7	Biolegend (100706)	0.3125:100
BV605	CD86	PO3	Biolegend (105125)	2.5:100
BV785	F4/80	BM8	Biolegend (123141)	2:100
BV605	Ki67	16A8	Biolegend (652413)	1.25:100
AF700	Ki67	16A8	Biolegend (652420)	0.25:100
BV421	MHC-II	M5/114.15.2	Biolegend (107632)	0.75:100
AF594	MHC-II	M5/114.15.2	Biolegend (107650)	2.5:100
BV785	PD-1	29F.1A12	Biolegend (135225)	1.25:100
PE	Tcf1	S33-966	Fisher Scientific (BDB564217)	1.25:100
BV711	Tim-3	RMT3-23	Biolegend (119727)	1.25:100
BV605	Tim-3	RMT3-23	Biolegend (119721)	1.25:100
Zombie Aqua (Viability)			Biolegend (423102)	0.5:100

Supporting Table T1: Antibodies used for flow cytometry.
